# Broad Substrate Specificity and High Catalytic Activity of *Sphingomonadaceae* PhoK-Type Phosphatases Implicated in Flame-Retardant Degradation

**DOI:** 10.1021/acsomega.5c05616

**Published:** 2025-08-29

**Authors:** Landry Freeman, Andrew Davis, Harley Gossen, Jake Estes, Andrew N. Bigley

**Affiliations:** Department of Chemistry and Physics, 8452 Southwestern Oklahoma State University, Weatherford, Oklahoma 73096, United States

## Abstract

The first two enzymes recognized to be PhoK-type phosphatases were from *Sphingobium* sp. TCM1 (*Sb*-PhoK) and *Sphingomonas* sp. BASR1 (*Sm*-PhoK) which were utilized in bioremediation of organophosphate flame-retardants and heavy metal contamination, respectively. The PhoK-type phosphatases are members of the nucleotide pyrophosphatase/phosphodiesterase (NPP) family of diesterases that have evolved a phosphatase activity. These enzymes were noted for very high activity with model compounds compared to other alkaline phosphatases, but very little was known about their substrate specificity or the activity with phosphomonoesters derived from flame-retardants or other environmental phosphoesters. Bioinformatics analysis has been utilized to identify PhoK homologues from a large group of *Sphingomonadaceae* family members including additional species that are known or suspected to utilize the organophosphate flame-retardants as nutrient sources. Nine homologues were selected for kinetic characterization using a synthesized library of organophosphate monoesters derived from flame-retardants, environmental phosphoesters, and biological monophosphates. The *Sphingomonadaceae* PhoK enzymes were found to have high enzymatic efficiency against a broad range of substrates. Against phenyl phosphate *Sm*-PhoK has a *k*_cat_ of 1100 s^–1^ and a *k*_cat_/*K*_m_ of 1.8 × 10^6^ M^–1^ s^–1^. The best overall activity was observed with the homologue from *Sphingobium yanoikuyae* (*Sy*-PhoK), another species known to degrade organophosphate flame-retardants. This enzyme hydrolyzed all tested substrates with an efficiency greater than 3 × 10^4^ M^–1^ s^–1^. The high catalytic activity and remarkably broad substrate specificity make the *Sphingomonadaceae* PhoK enzymes particularly suited for bioremediation as well as commercial applications where high turnover will be advantageous.

## Graphical abstract

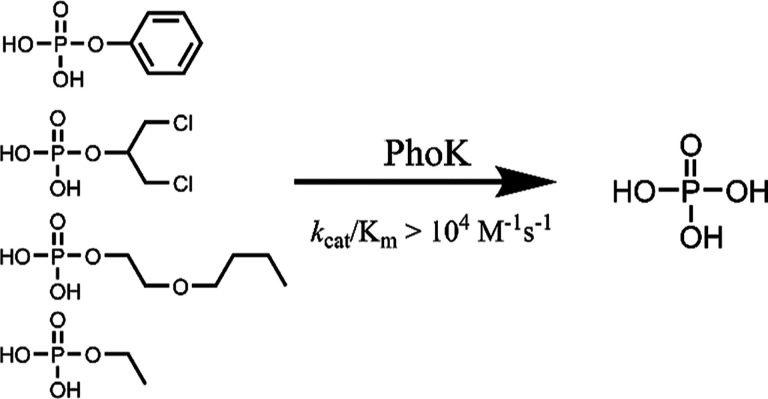

## Introduction

The organophosphate flame-retardants are phosphate triesters included in plastics and durable foam products to limit flammability.^1^ These compounds have only been widely used since the early 2000s when they began to replace the polybrominated diphenyl ether flame-retardants.^2^ The organophosphate flame-retardants are currently used at rates of hundreds of tons per year. Unfortunately, when these compounds such as triphenyl phosphate, tris-1,3-dichloroisopropyl phosphate and tris-2-butoxyethyl phosphate leach out of plastics, they are carcinogens, developmental toxins, and endocrine disruptors.^1^ Despite their recent introduction into the environment, the bacteria *Sphingobium* sp. TCM1 was found to have evolved a three-step hydrolytic pathway to utilize the phosphate from the flame-retardants as a nutrient source^3–5^ (Figure 1).

**Figure 1:**
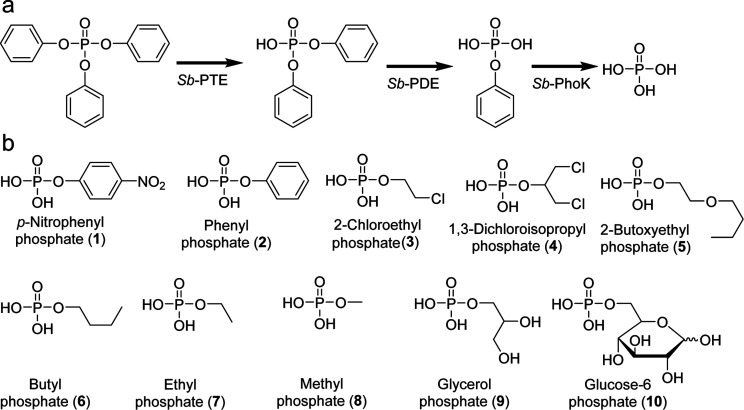
(a) Metabolic pathway for organophosphate flame-retardant degradation evolved in *Sphingobium* sp. TCM1. (b) Phosphomonoesters tested in this work.

The third and final step in the pathway in *Sphingobium* sp. TCM1 was found to be catalyzed by the PhoK-type phosphatase *Sb*-PhoK.^5,6^ The PhoK-type phosphatases are members of the nucleotide pyrophosphatase/phosphodiesterase (NPP) family of enzymes.^7^ The NPP-family is part of the larger alkaline phosphatase (AP) super family, but the NPP-family enzymes are diesterases rather than the typical phosphatases seen in the AP-super family.^8^ The key distinction between the NPP-family and the AP-super family is thought to lie in the metal centers of the enzymes. Both families contain a binuclear metal center and a serine/threonine which serves as the initial nucleophile in a double displacement mechanism, but the AP-super family contains a third metal that stabilizes the second negatively charged oxygen in the monoester substrates. The NPP-family is specific for diesters which lack the second charged oxygen, and they have lost the third metal site in the course of evolution. Despite being members of the NPP-family, the PhoK-type phosphatases appear to have reverted back to the phosphatase reaction. The prototype of the PhoK-type phosphatases was first identified in *Sphingomonas* sp. BSAR-1 (*Sm*-PhoK) where it was used in the bioremediation of Uranium contaminated water.^9^ The crystal structure of *Sm*-PhoK (pdb: 5xwk and 3q3q) revealed that it contains the binuclear metal center of the NPP-family and a threonine nucleophile. The overall fold consists of a large central αβ-sandwich domain and smaller αβ-fold domain^7^ (Figure S1). The active site is located at the C-terminal end of the central β-sheet of the large domain. The α-metal is ligated by residues D49, T89, D345 and H346 and the β-metal is ligated by D300, H304, and H491 (Figure S1 B). The reversion to the phosphatase reaction is proposed to have been facilitated by the introduction of a lysine residue in the active site that occupies the same site where the third metal is found in the AP-super family^7^ (Figure S1 C). This lysine (K171) along with R173 and N110 hydrogen bond to the second charged oxygen in the phosphomonoester substrate. The high enzymatic activity and potential utility in bioremediation make the PhoK-type phosphatases of significant interest, but very little is currently known about their catalytic activity. The crystal structure of *Sm*-PhoK was solved, and it was noted that the enzyme has high specific activity relative to the *Escherichia coli* alkaline phosphatase, but the substrate specificity of the enzyme was never determined.^7^ *Sb*-PhoK was shown to allow growth on tris-2-chloroethyl phosphate and that expression was induced when tris-2-chloroethyl phosphate was the sole phosphate source, but *Sb*-PhoK has never been characterized with phosphate monoesters derived from the flame-retardants, presumably due to a lack of a commercial source for these compounds.^5,6^ Only two other examples of PhoK-type phosphatases have been reported in the literature neither of which have been kinetically characterized.^10,11^ To meet these challenges, we used bioinformatics analysis to identify homologues of *Sm*- and *Sb*-PhoK, and synthesized a library of flame-retardant derived and environmental phosphoesters (Figure 1B). A total of nine PhoK-type phosphatases from the *Sphingomonadaceae* bacterial family were selected for characterization including enzymes from three additional species known to degrade organophosphate flame-retardants.

## Results and Disscussion

To identify additional PhoK-type phosphatases, the sequence for *Sb*-PhoK was used as the input sequence for the Enzyme Function Initiative-Enzyme Similarity Tool with a cutoff of *E*-value of 10^–20^.^12^ The 1928 homologues identified were then used to construct a sequence similarity network with a maximum *E*-value of 10^–40^. When the network is viewed with an alignment score cutoff of 175 (∼50% identity), the homologues cluster primarily according to genus with the homologues from the *Sphingomonadaceae* family clustering together (Figure 2). This cluster contained the sequences for PhoK homologues from *Sphingobium* sp. TCM1 (*Sb*-PhoK), *Sphingomonas* sp. TDK1 (*Sm*TDK1-PhoK), *Sphingopyxis terrae* (*St*-PhoK) and *Sphingobium yanoikuyae* (*Sy*-PhoK), which are all known degraders of organophosphate flame-retardants.^4,13,14^ Also located in this cluster were PhoK homologues from *Novosphingobium aromaticivorans* (*Na*-PhoK), *Novosphingobium* sp. EMRT2 (*No*-PhoK), and *Sphingomonas* sp. BSAR-1 (*Sm*-PhoK), which are suspected degraders of organophosphate flame-retardants based on the species association with contaminated sites.^9,15,16^ For comparison two additional sequences from *Novosphingobium guangzhouense* (*Ng*-PhoK), a species isolated from refinery soil in China, and *Sphingomonas* sp. SRS2 (*Sm*SRS2-PhoK), a species isolated from herbicide treated agricultural soil, were selected for characterization.^17,18^ Neither of these species has a known association with organophosphate flame-retardants. *Ng*-PhoK was selected from the main *Sphingomonadaceae* PhoK cluster, and *Sm*SRS2-PhoK was selected from a smaller PhoK cluster containing *Sphingomonas* species.

**Figure 2:**
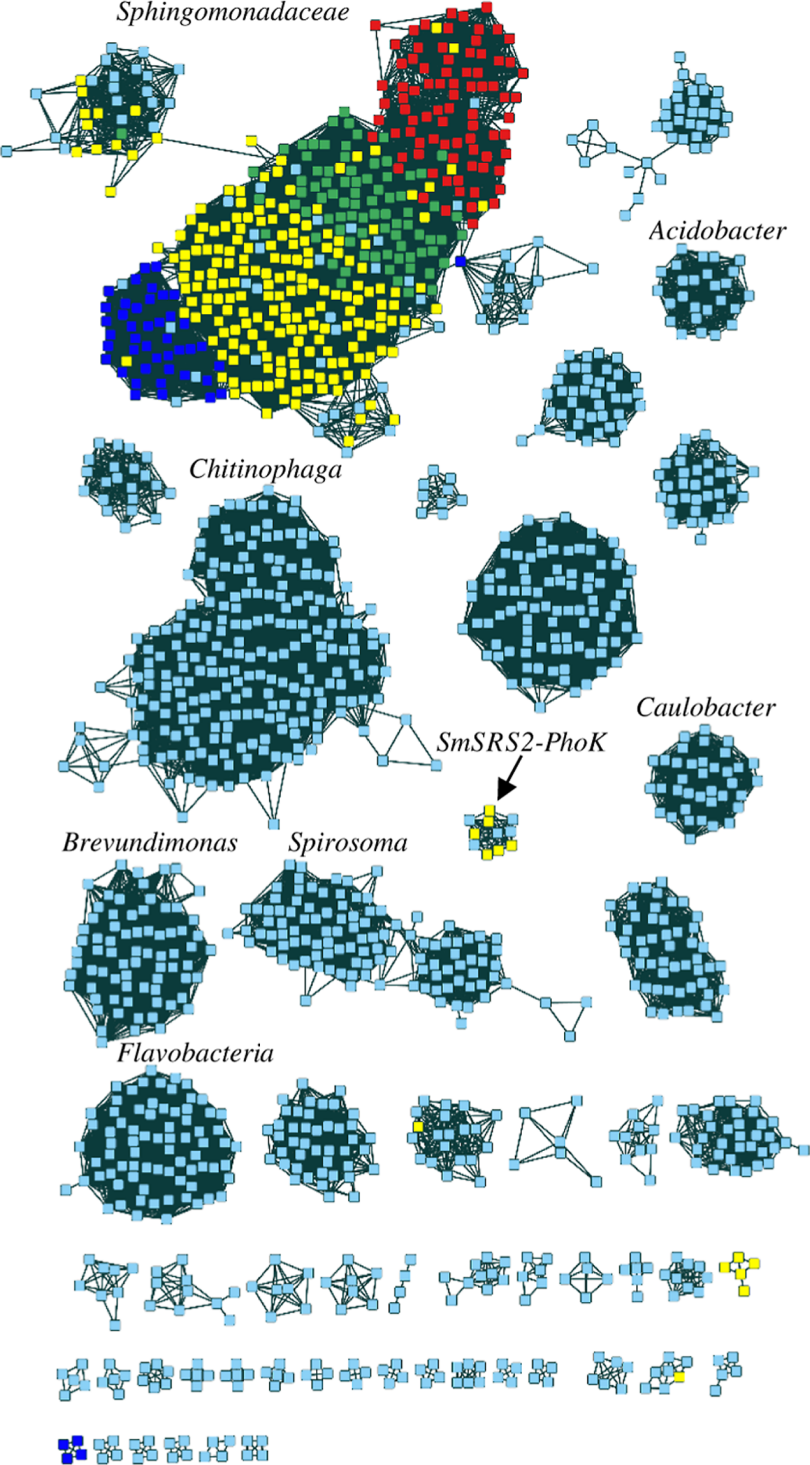
Sequence similarity network of PhoK-homologues shown with an alignment score cutoff of 175. Sequences primarily cluster according to genus with the members of the *Sphingomonadaceae* family (*Sphingopyxis* (dark blue), *Sphingomonas* (yellow), *Sphingobium* (green), and *Novosphingobium* (red) clustering together). Other clusters are identified by the primary genus found in the cluster. The characterized protein *Sm*-SRS2-PhoK not found in the *Sphingomonadaceae* cluster is labeled. Singlets and clusters with less than 4 sequences have been omitted for clarity.

The genes for the nine selected homologues were purchased as synthetic constructs and expressed in *E. coli* Bl21­(DE3) cells. All proteins were successfully purified with yields of ∼10 mg/L of culture. Sequence alignments with *Sm*-PhoK confirmed that the metal center, the threonine which serves as the nucleophile, and active site lysine (K171), arginine (R173), and asparagine (N110) are all conserved in the homologues selected for characterization (Figure S2). Metal analysis found ∼2 zinc per protein, and it was found that addition of excess zinc did not increase the rate of reaction for any homologues (Table S1). All homologues were characterized with the model compound *p*-nitrophenyl phosphate (**1**) and the flame-retardant derived monoesters phenyl phosphate (**2**), 2-chloroethyl phosphate (**3**), 1,3-dichloroisopropyl phosphate (**4**), and 2-butoxyethyl phosphate (**5**) as well as butyl phosphate (**6**) which is a break down product of tributyl phosphate which is used in nuclear fuel processing. The homologues were also tested with ethyl phosphate (**7**) and methyl phosphate (**8**) which are common environmental organophosphates from the breakdown of organophosphate insecticides and two biological phosphoesters, glycerol phosphate (**9**) and glucose-6-phosphate (**10**). (Figure 1).

The homologues were characterized with *p*-nitrophenyl phosphate and phenyl phosphate using standard UV/vis spectroscopy to monitor the reaction (Table 1 and Figure S3). Exact experimental conditions are given in Table S2. The *k*_cat_ values for these two compounds were similar for the homologues with only *No*-PhoK showing more than a 2-fold reduction in *k*_cat_ with the phenyl leaving group compared to the highly activated *p*-nitrophenyl group suggesting that the hydrolysis of the covalent intermediate might be the rate limiting step of the reaction. *Sm*-PhoK demonstrated the highest *k*_cat_ with a rate greater than 1000 s^–1^ for both compounds. The *K*_m_ values for the homologues tended to be somewhat higher for phenyl phosphate resulting in a slightly lower efficiency for most homologues. A noted exception to that trend was seen with *St*-PhoK which had *K*_m_ values within error of each other for both the *p*-nitrophenyl and phenyl leaving group. The most efficient enzyme with *p*-nitrophenyl phosphate was *Sm*-PhoK (*k*_cat_/*K*_m_ = 7.9 × 10^6^ M^–1^ s^–1^) while *St*-PhoK was the best with phenyl phosphate (*k*_cat_/*K*_m_ = 3.9 × 10^6^ M^–1^ s^–1^) though the activity of the other homologues was still very good with values in excess of 10^5^ M^–1^ s^–1^. Interestingly the two homologues that are not associated with organophosphate flame-retardant degradation also showed significant activity against these compounds. *Ng*-PhoK which is in the same cluster as the flame-retardant associated homologues showed activity on par with the others. *Sm*SRS2-PhoK which was found in a smaller cluster of homologues showed a significant decrease in *k*_cat_ value compared to the others (*k*_cat_ = 17 vs >200 s^–1^).

**Table 1:** Kinetic Constants with Model Substrate *p*-Nitrophenyl Phosphate and Flame-Retardant Derived Substrate Phenyl Phosphate

compound	*p*-nitrophenyl phosphate (1)			phenyl phosphate (2)		
enzyme	*k*_cat_ (s^–1^)	*K*_m_ (μM)	*k*_cat_/*K*_m_ (M^–1^ s^–1^)	*k*_cat_ (s^–1^)	*K*_m_ (μM)	*k*_cat_/*K*_m_ (M^–1^ s^–1^)
*Sb*-PhoK	416 ± 3	69 ± 2	6.1 ± 0.2 × 10^6^	460 ± 10	650 ± 30	7.1 ± 0.4 × 10^5^
*Sm*TDK1-PhoK	460 ± 5	450 ± 10	1.02 ± 0.03 × 10^6^	365 ± 7	600 ± 20	6.1 ± 0.3 × 10^5^
*St*-PhoK	440 ± 5	78 ± 3	5.7 ± 0.2 × 10^6^	314 ± 6	81 ± 5	3.9 ± 0.2 × 10^6^
*Sy*-PhoK	1230 ± 10	176 ± 4	7.0 ± 0.2 × 10^6^	930 ± 30	670 ± 40	1.4 ± 0.1 × 10^6^
*Na*-PhoK	303 ± 3	89 ± 3	3.4 ± 0.1 × 10^6^	336 ± 6	390 ± 20	8.7 ± 0.4 × 10^5^
*No*-PhoK	621 ± 6	153 ± 5	4.1 ± 1.1 × 10^6^	259 ± 6	300 ± 20	8.7 ± 0.6 × 10^5^
*Sm*-PhoK	1370 ± 10	174 ± 4	7.9 ± 0.2 × 10^6^	1100 ± 10	600 ± 20	1.84 ± 0.06 × 10^6^
*Ng*-PhoK	297 ± 4	90 ± 4	3.3 ± 0.1 × 10^6^	190 ± 3	250 ± 9	7.5 ± 0.3 × 10^5^
*Sm*SRS2-PhoK	12.0 ± 0.1	88 ± 4	1.36 ± 0.07 × 10^5^	16.6 ± 0.2	104 ± 5	1.59 ± 0.07 × 10^5^

The remaining compounds tested lack a chromogenic leaving group. To follow these reactions ^31^P NMR was used in total hydrolysis reactions to determine the *k*_cat_/*K*_m_ for the reaction under pseudo first order conditions. The total phosphorus signal was integrated to determine the fraction hydrolyzed as a function of time which was fit to a single exponential (Figures 3 and S5–S19). Exact experimental conditions are given in Table S3. The activity against compounds **3**–**10** was reduced compared to the activity seen with compounds **1** and **2** which contained aromatic leaving groups though most compounds were hydrolyzed with efficiencies greater than 10^4^ M^–1^ s^–1^ (Table 2). Compounds **3**–**10** represent chlorinated alkyls, ethers, simple alkyls, and carbohydrate substrates, but there is very little specificity observed with the *Sphingomonadaceae* enzymes. *Sy*-PhoK showed the best overall activity with enzymatic efficiency of >3 × 10^4^ M^–1^ s^–1^ for all compounds tested. With this homologue 1,3-dichloroisopropyl phosphate (**4**) was the best substrate with a *k*_cat_/*K*_m_ of 1 × 10^5^ M^–1^ s^–1^ and its worst substrate was butyl phosphate (**6**) which was reduced 3-fold. *Ng*-PhoK demonstrated the largest selectivity with the best substrate being 2-chloroethyl phosphate (2) (*k*_cat_/*K*_m_ = 3.8 × 10^4^ M^–1^ s^–1^) and the worst being ethyl phosphate (*k*_cat_/*K*_m_ = 5.5 × 10^3^ M^–1^ s^–1^). The other homologues all showed less selectivity with *St*-PhoK only showing 2-fold difference between the best and worst substrates.

**Figure 3:**
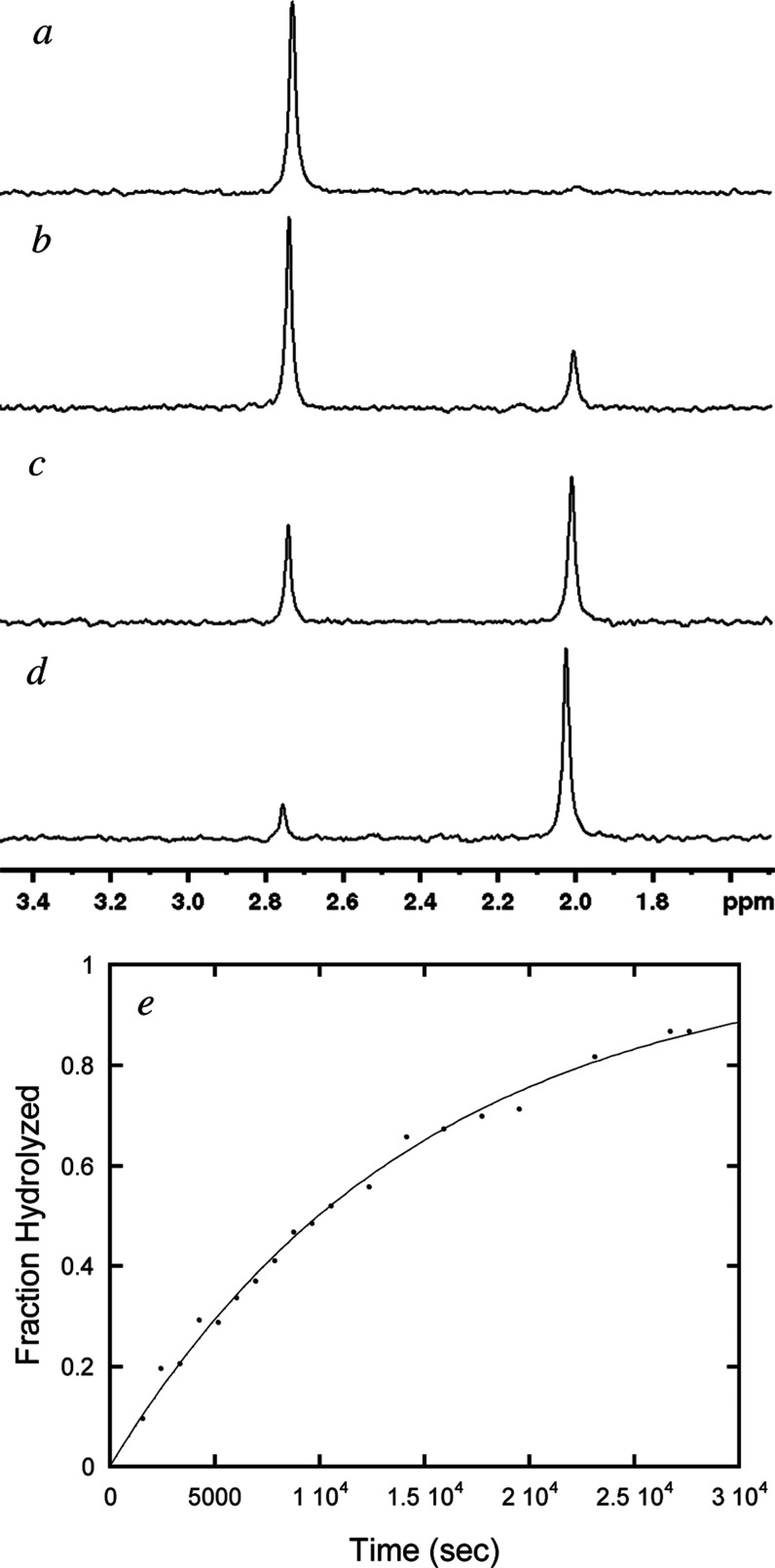
Hydrolysis of 2.5 mM 1,3-dichloroisopropyl phosphate (**4**) by 4.1 nM *Sb*-PhoK followed by ^31^P NMR. Panel (a) shows the ^31^P NMR spectra 1,3-dichloroisopropyl phosphate (**4**) with resonance at 2.78 ppm before addition of enzyme. Panel (b) shows appearance of the phosphate product at 2.01 ppm 70 min after addition of enzyme. Panel (c) shows reaction after 235 min, and panel (d) shows product after 460 min. Panel (e) shows exponential curve fit to NMR data.

**Table 2:** Enzymatic Efficiency (*K*_cat_/*K*_m_) for Homologs with Flame-Retardant Derived Phosphoesters and Environmental Organophosphates (M^–1^ s^–1^)­^a^

	compound							
enzyme	**3**	**4**	**5**	**6**	**7**	**8**	**9**	**10**
*Sb*-PhoK	3.6 × 10^4^	1.7 × 10^4^	2.9 × 10^4^	1.3 × 10^4^	2.7 × 10^4^	1.2 × 10^4^	2.5 × 10^4^	2.2 × 10^4^
*Sm*TDK1-PhoK	3.6 × 10^4^	5.4 × 10^4^	1.9 × 10^4^	4.2 × 10^3^	1.1 × 10^4^	2.0 × 10^4^	2.0 × 10^4^	1.1 × 10^4^
*Sp*-PhoK	2.8 × 10^4^	2.3 × 10^4^	2.7 × 10^4^	1.2 × 10^4^	1.7 × 10^4^	1.8 × 10^4^	2.3 × 10^4^	2.2 × 10^4^
*Sy*-PhoK	7.1 × 10^4^	1.0 × 10^5^	7.6 × 10^4^	3.1 × 10^4^	4.8 × 10^4^	4.1 × 10^4^	5.9 × 10^4^	4.8 × 10^4^
*Na*-PhoK	4.1 × 10^4^	1.1 × 10^4^	3.2 × 10^4^	1.4 × 10^4^	1.5 × 10^4^	1.1 × 10^4^	1.9 × 10^4^	1.8 × 10^4^
*No*-PhoK	6.2 × 10^4^	1.9 × 10^4^	5.6 × 10^4^	1.7 × 10^4^	2.0 × 10^4^	1.6 × 10^4^	2.4 × 10^4^	1.1 × 10^4^
*Sm*-PhoK	8.4 × 10^4^	4.5 × 10^4^	7.9 × 10^4^	2.4 × 10^4^	2.9 × 10^4^	4.8 × 10^4^	5.7 × 10^4^	3.9 × 10^4^
*Ng*-PhoK	3.8 × 10^4^	3.7 × 10^4^	2.7 × 10^4^	1.2 × 10^4^	5.5 × 10^3^	1.5 × 10^4^	1.9 × 10^4^	2.1 × 10^4^
*Sm*SRS-PhoK	2.1 × 10^3^	1.0 × 10^3^	1.3 × 10^3^	4.4 × 10^2^	4.4 × 10^2^	4.5 × 10^2^	1.6 × 10^3^	9.7 × 10^2^

a Errors from curve fit were generally less than 10% and are reported in Supporting Information Table.

The *Sphingomonadaceae* PhoK homologues show remarkably similar activity across this broad range of substrates. *Sm*SRS2 showed activity diminished between 10- and 100-fold compared to the other homologues, but the activity of all of the homologues identified in the main group of *Sphingomonadaceae* enzymes are all quite similar between the various substrates. The substrate with the biggest difference between homologues was 1,3-dichloroisopropyl phosphate what had a 9-fold difference between *Sy*-PhoK and *Na*-PhoK (*k*_cat_/*K*_m_ = 1.0 × 10^5^ M^–1^ s^–1^ vs 1.1 × 10^4^ M^–1^ s^–1^), but for 2-chloroethyl phosphate (**3**), ethyl phosphate (**7**) and glycerol phosphate (**9**) the difference between the most efficient and least efficient homologue was only about 3-fold.

The high activity and broad specificity of these enzymes is quite remarkable. The first PhoK enzyme identified was *Sm*-PhoK which was identified during efforts to facilitate bioremediation of heavy metal contamination.^9^ *Sb*-PhoK was identified as being involved in the biological degradation of the organophosphate flame-retardants.^5^ We have now shown that the *Sphingomonadaceae* family PhoK-type phosphatases are broad specificity phosphatases with remarkably high enzymatic efficiency against a wide range of substrates as well as *k*_cat_ values much higher than the standard *E. coli* alkaline phosphatase.^5^ These enzymes have significant potential in both bioremediation efforts as well as potential commercial applications where broad specificity and high catalytic activity will be a significant advantage.

## Materials and Methods

In general lab supplies and chemicals were from Fisher Scientific. Silica gel (60 mesh) and reagents for the synthesis of compounds were from Sigma-Aldrich and were generally ACS grade or the highest quality available. Solvents used were from Fisher Scientific or Pharmco. Centrifugation was conducted using a Sorvall Lynx 4000 High Speed Centrifuge equipped with a F12-6x500 LEX rotor. NMR spectra were recorded on a Bruker 300 MHz Avance III spectrometer.

### Compounds Tested

The compounds tested included *p*-nitrophenyl phosphate (**1**), a common model compound for phosphatase reaction,^8^ phenyl phosphate, 2-chloroethyl phosphate, 1,3-dichloroisopropyl phosphate, 2-butoxyethyl phosphate, the resultant monoesters from the breakdown of the corresponding tris-ester flame-retardants, which are known or suspected carcinogens, developmental toxins, and endocrine disruptors.^19–23^ Also tested was butyl phosphate, the monoester resulting from the degradation of tributyl phosphate which is used in nuclear fuel processing,^24^ and ethyl and methyl phosphate which are common environmental phosphate esters that result from the degradation of organophosphate insecticides.^25^ *p*-Nitrophenyl phosphate (**1**) phenyl phosphate (**2**), glycerol phosphate (α-, β- mixed isomers) (**9**), and glucose-6-phosphate (**10**) were purchased from Sigma-Aldrich. The remaining compounds were synthesized by condensation of trichlorophosphate with the corresponding alcohol followed by hydrolysis to the phosphate using modifications of previously described methods.^26^ The dichlorophosphate precursor for ethyl phosphate (**7**) and methyl phosphate (**8**) was purchased from Sigma-Aldrich.

### Synthesis of 2-Chloroethyl Dichlorophosphate

Trichlorophosphate (2.6 mL, 4.28 g, 28 mmol, 1 equiv) was added to 100 mL diethyl ether and chilled to 0 °C on ice with stirring. To this mixture 2-chloroethanol (1.9 mL, 1.92 g, 25 mmol, 0.9 equiv) was added followed by triethyl amine (3.9 mL, 2.83 g, 28 mmol, 1 equiv). Addition of the triethyl amine caused a rapid precipitation of a white solid. The reaction vessel was purged with nitrogen, allowed to warm to room temperature and stirred overnight. Following filtration to remove the solid, the solvent was removed under reduced pressure rotary evaporation leaving a yellow oil as the crude product. TLC identification of product was done using a 3:1 ratio of hexanes/ethyl acetate with plates stained with I_2_. Crude product was purified by silica gel chromatography. The crude product was loaded on the column in hexanes and washed with 100 mL of hexanes followed by elution with 3:1 hexanes/ethyl acetate. Solvent was removed yielding 1.98 g (42% yield) of 93% pure product as a yellow oil.

^1^H NMR (300.13 MHz, CDCl_3_): δ 4.60–4.52 (d, t *J* = 9.45 Hz, *J* = 5.72 Hz, 2H), 3.83–3.80 (t, *J* = 5.72 Hz, 2H).

^31^P NMR (121.49 MHz, CDCl_3_, proton coupled): δ 8.00–7.85 (t, *J* = 9.45 Hz).

### Synthesis of 2-Chloroethyl Phosphate (**3**)

2-Chloroethyl dichlorophosphate (0.789 g, 4.03 mmol, 1 equiv) was dissolved in 4.03 mL of THF and chilled to 0 °C in an ice bath. Once chilled, 4.03 mL of 2 M aqueous NaOH (2 equiv) was added dropwise and the reaction stirred for 2 h at 0 °C. The solvent was removed by reduced pressure rotary evaporation leaving a wet white solid. The solid was extracted with diethyl ether. Following filtration, the solvent was removed yielding 0.503 g (78%) of yellow oil as the pure product.

^1^H NMR (300.13 MHz, CDCl_3_): δ 3.78–3.71 (q *J* = 7.0 Hz, 2H), 1.29–1.24 (t, *J* = 5.70 Hz, 2H).

^31^P NMR (121.49 MHz, D_2_O, proton coupled): δ 0.10 to −0.02 (t, *J* = 7.11 Hz).

### Synthesis of 1,3-Dichloroisopropyl Dichlorophosphate

Trichlorophosphate (2.6 mL, 4.28 g, 28 mmol, 1 equiv) was added to 100 mL diethyl ether and chilled to 0 °C on ice with stirring. To this mixture 1,3-dichloro-2-propanol (2.26 mL, 3.08 g, 25 mmol, 0.9 equiv) was added followed by triethyl amine (3.9 mL, 2.83 g, 28 mmol, 1 equiv). Addition of the triethyl amine caused a rapid precipitation of a white solid. The reaction was stirred for 2 h in an ice bath. Following filtration to remove the solid, the solvent was removed under reduced pressure rotary evaporation leaving a yellow oil as the crude product. Crude product was loaded on a silica gel column in hexanes washed with 100 mL of hexanes and eluted with 3:1 hexanes/ethyl acetate. Solvent was removed yielding 1.84 g (30% yield) of 94% pure product as a yellow oil.

^1^H NMR (300.13 MHz, CDCl_3_): δ 5.09–4.98 (d, qu *J* = 11.10 Hz, *J* = 5.04 Hz, 1H), 3.85–3.83 (d, *J* = 5.04 Hz, 4H).

^31^P NMR (121.49 MHz, CDCl_3_, proton coupled): δ 8.19–8.1 (d, *J* = 11.10 Hz).

### Synthesis of 1,3-Dichloroisopropyl Phosphate (**4**)

THF (7.47 mL) was chilled in an ice bath, and 1,3-dichloroisopropyl phosphate (1.84 g, 7.47 mmol, 1 equiv) was added. Once chilled, 7.47 mL of 2 M aqueous NaOH (2 equiv) was added dropwise and the reaction stirred for 2 h at 0 °C. The solvent was removed by reduced pressure rotary evaporation leaving a wet white solid. The solid was extracted with diethyl ether. Following filtration, the solvent was removed yielding 1.45 g (93% yield) of yellow oil as the pure product.

^1^H NMR (300.13 MHz, CDCl_3_): δ 4.72–4.62 (m, 1H), 3.86–3.85 (d, *J* = 5.04 Hz, 4H).

^31^P NMR (121.49 MHz, CDCl_3_): δ −0.99 (s).

^31^P NMR (121.49 MHz, D_2_O, proton coupled): δ −0.82 to −0.90 (d, *J* = 8.89 Hz).

### Synthesis of 2-Butoxyethyl Dichlorophosphate

Trichlorophosphate (2.6 mL, 4.28 g, 28 mmol, 1 equiv) was added to 100 mL diethyl ether and chilled to 0 °C on ice with stirring. To this mixture 2-butoxyethanol (3.41 mL, 1.78 g, 26 mmol, 0.9 equiv) was added followed by triethyl amine (3.9 mL, 3.07 g, 28 mmol, 1 equiv). Addition of the triethyl amine caused a rapid precipitation of a white solid. The reaction vessel was allowed to warm to room temperature and stirred overnight. Following filtration to remove the solid, the solvent was removed under reduced pressure rotary evaporation leaving a yellow oil as the crude product. Product could be visualized on TLC using I_2_ followed by Hessian’s stain without heating. Crude product was purified by silica gel chromatography. Crude product was loaded on column in hexanes washed with 100 mL of hexanes and eluted with 3:1 hexanes/ethyl acetate. Solvent was removed yielding 0.799 g (14% yield) as a yellow oil.

^1^H NMR (300.13 MHz, CDCl_3_): δ 4.49–4.43 (m, 2H), 3.77–3.74 (m, 2H), 3.54–3.50 (t, *J* = 6.50 Hz, 2H), 1.64–1.57 (m, 2H), 1.46–1.28 (m, 2H), 0.97–0.92 (t, *J* = 7.35 Hz, 3H).

^31^P NMR (121.49 MHz, CDCl_3_, proton coupled): δ 7.87 (t, *J* = 9.36 Hz).

### Synthesis of 2-Butoxyethyl Phosphate (**5**)

2-Butoxyethyl dichlorophosphate (0.799 g, 3.4 mmol, 1 equiv) was dissolved in 3.4 mL of THF and chilled to 0 °C in an ice bath. Once chilled, 3.4 mL of 2 M aqueous NaOH (2 equiv) was added dropwise and the reaction stirred for 2 h at 0 °C. The solvent was removed by reduced pressure rotary evaporation leaving a wet white solid. The solid was extracted with diethyl ether. Following filtration, the solvent was removed yielding 0.761 g (66%) of yellow oil as the pure product.

^1^H NMR (300.13 MHz, CDCl_3_): δ 4.21–4.15 (m, 2H), 3.72–3.69 (m, 2H), 3.57–3.52 (t, *J* = 6.77 Hz, 2H), 1.64–1.55 (m, 2H), 1.45–1.31 (m, 2H), 0.96–0.91 (t, *J* = 7.32 Hz, 3H).

^31^P NMR (121.49 MHz, D_2_O, proton coupled): δ 3.61–3.52 (t, *J* = 5.43 Hz).

### Synthesis of Butyl Dichlorophosphate

Trichlorophosphate (2.6 mL, 4.28 g, 28 mmol, 1 equiv) was added to 100 mL diethyl ether and chilled to 0 °C on ice with stirring. To this mixture n-butanol (2.2 mL, 1.78 g, 25 mmol, 0.9 equiv) was added followed by triethyl amine (3.9 mL, 2.83 g, 28 mmol, 1 equiv). Addition of the triethyl amine caused a rapid precipitation of a white solid. The reaction vessel was purged with nitrogen, allowed to warm to room temperature and stirred overnight. Following filtration to remove the solid, the solvent was removed under reduced pressure rotary evaporation leaving a yellow oil as the crude product. Crude product was purified by silica gel chromatography. Crude product was loaded on column in hexanes washed with 100 mL of hexanes and eluted with 3:1 hexanes/ethyl acetate. Solvent was removed yielding 2.84 g (62% yield) as a yellow oil.

^1^H NMR (300.13 MHz, CDCl_3_): δ 4.41–4.33 (m, 2H), 1.87–1.77 (qu, *J* = 6.98 Hz, 2H), 1.56–1.44 (sex, *J* = 7.44 Hz, 2H), 1.02–0.97 (t, *J* = 7.34 Hz, 3H).

^31^P NMR (121.49 MHz, CDCl_3_, proton coupled): δ 7.13–6.98 (t, *J* = 8.49 Hz).

### Synthesis of Butyl Phosphate (**6**)

Butyl dichlorophosphate (1.42 g, 7.43 mmol, 1 equiv) was dissolved in 7.43 mL of THF and chilled to 0 °C in an ice bath. Once chilled, 7.43 mL of 2 M aqueous NaOH (2 equiv) was added dropwise and the reaction stirred for 2 h at 0 °C. The solvent was removed by reduced pressure rotary evaporation leaving a wet white solid. The solid was extracted with diethyl ether. Following filtration, the solvent was removed yielding 0.761 g (66%) of yellow oil as the pure product.

^1^H NMR (300.13 MHz, CDCl_3_): δ 4.41–4.04 (q *J* = 6.64 Hz, 2H), 1.73–1.64 (m, 2H), 1.49–1.37 (m, 2H), 0.97–0.92 (t, *J* = 7.35 Hz, 3H).

^31^P NMR (121.49 MHz, D_2_O, proton coupled): δ 0.46–0.35 (t, *J* = 7.65 Hz).

### Synthesis of Ethyl Phosphate (**7**)

THF (9.34 mL) was chilled in an ice bath and ethyl dichlorophosphate (1.52 g, 9.34 mmol, 1 equiv) was added. Once chilled, 9.34 mL of 2 M aqueous NaOH (2 equiv) was added dropwise. The reaction was stirred on ice for 2 h. The solvent was removed by reduced pressure rotary evaporation leaving a wet white solid. The solid was extracted with diethyl ether. Following filtration, the solvent was removed yielding 0.665 g (57% yield) of yellow oil as the pure product.

^1^H NMR (300.13 MHz, CDCl_3_): δ 3.53–3.50 (d, *J* = 11.14 Hz 3H).

^31^P NMR (121.49 MHz, MeOD, proton coupled): δ −0.26 to −0.54 (q, *J* = 11.24 Hz).

### Synthesis of Methyl Phosphate (**8**)

Methyl dichlorophosphate (1 g, 6.7 mmol, 1 equiv) was added to 6.7 mL of THF with stirring and chilled on ice. To this solution 6.7 mL of 2 M NaOH (2 equiv) was added dropwise and the reaction allowed to stir on ice for 2 h. Solvent was removed via reduced pressure rotary evaporation yielding a mixture of oil and solid. Diethyl ether (20 mL) added to dissolve the product and a small amount of anhydrous sodium sulfate was added to dry. Organic phase was transferred to a round-bottom flask and the solvent removed via reduced pressure rotary evaporation. The residual oil was dissolved in minimal methanol and transferred to a vial. After removing solvent via vacuum the final product (548 mg, 73% yield) was recovered as a pale-yellow oil.

^1^H NMR (300.13 MHz, DMSO-*d*_6_): δ 4.19–4.15 (m, 2H), 1.40–1.36 (t, *J* = 6.75 Hz, 3H).

^31^P NMR (121.49 Hz, D_2_O, proton coupled): δ 0.34–0.22 (t, *J* = 7.29 Hz).

### Bioinformatics Analysis

*Sb*-PhoK was used as a search input for the Enzyme Function Initiative-Enzyme Similarity Tool to identify evolutionarily related sequences with a maximum *E*-value of 10^–20^.^12,27,28^ The 1928 sequences identified were used to construct a sequence similarity network with an edge defined as an *E*-value of 10^–40^ or less. The resultant network contained a total of 1,361,826 evolutionary relationships among the 1928 homologues. The final network was visualized with Cytoscape using an alignment score cutoff of 175.^29^ The view was generated with the embedded j-files organic layout.

### Sequence Alignments and Structure Analysis

Sequence alignments were done with the Clustal W algorithm in SnapGene software from Dotmatrix. The crystal structures were analyzed and figures prepared from pdb: 3q3q and pdb: 5xwk using the program UCSF Chimera.^30^ Signal peptides were predicted using the SignalP 5.0 server.^31^ All proteins were found to contain Sec/SPI cleavage sites. Molecular weights and extinction coefficients for all proteins were calculated using SnapGene for the proteins lacking the signal peptides.

### Protein Expression and Purification

The genes for *Sb*-PhoK, *Sm*TDK-PhoK, *Sp*-PhoK, *Sy*-PhoK, *Na*-PhoK, *No*-PhoK, *Sm*-PhoK, *Ng*-PhoK and *Sm*SRS-PhoK were purchased as synthetic DNA optimized for expression in *E. coli* from Twist Bioscience. All genes were purchased as colonial genes inserted into pET29a between the NdeI and XhoI cut sites which fused a 6xHisTag in frame with the proteins. Freshly transformed colonies of BL21­(DE3) cells were inoculated into 5 mL of LB broth supplemented with 50 μg/mL kanamycin and incubated at 37 °C overnight. For each protein two 1 L cultures of LB with 50 μg/mL kanamycin were inoculated with 1 mL of the overnight culture and grown at 37 °C until the OD_600_ reached 0.5. The temperature was reduced to 15 °C and protein expression was induced by addition of IPTG to 0.1 mM final concentration. Expression was allowed to proceed overnight (∼18 h) and cells were harvested by centrifugation for 10 min at 4250*g* in a Sorvall Lynx 4000 centrifuge equipped with a F12-6x500 LEX rotor. Cell pellets were stored at −80 °C prior to protein isolation. With the exception of *Sm*TDK-PhoK, the proteins were purified by Nickle affinity chromatography. Cells were resuspended in 40 mL of binding buffer (50 mM Hepes pH 8.0, 500 mM NaCl and 20 mM imidazole). Bovine pancreatic DNaseI (1 mg) from Sigma-Aldrich was added, and the cells were sonicated on ice for a total of 10 min (5 cycles of 2 min with 10 s on, 15 s off with 5 min rest between cycles) using a Fisher Scientific Sonic Dismembrator 500 set to 70% max amplitude. Cellular debris were removed by centrifugation at 10,000 rpm for 10 min. The resultant supernatant was passed through a 0.45 μm syringe filter and loaded on to a GE Healthcare ACTA Prime Plus FPLC with a 5 mL Cytiva His Trap HP column. The column was washed with 25 mL of binding buffer and the protein eluted with a 0–50% gradient of Elution Buffer (50 mM Hepes pH 8.0, 500 mM NaCl, and 500 mM Imidazole pH 8.0) over 100 mL. The flow rate for the FPLC was 5 mL/min except for the protein loading step which was lowered to 2.5 mL/min. The protein was collected in 5 mL fractions. Fractions showing activity against *p*-nitrophenyl phosphate were subjected to SDS-PAGE to determine purity using Minprotean any kDa Stain Free TGX precast gels from BioRad which were visualized on a Gel DocEZ imagining system from BioRad. Pure fractions were then dialyzed against 50 mM Hepes pH 8.0 to remove imidazol and salt using Spectra/Por 3 (3.5 MWCO) 45 mm dialysis tubing. *St*-PhoK was found to be unstable in the absence of salt, so it was dialyzed against 50 mM Hepes pH 8.0 with 500 mM NaCl. Proteins were concentrated to above 2 mg/mL using an Amicon Ultra 15 (Ultracel 10 K membrane) centrifugal ultrafiltration device. Aliquots of the concentrated proteins were flash frozen in liquid nitrogen for storage at −80 °C prior to kinetic testing. Protein concentration was determined by measuring the absorbance at 280 nm on a DeNovix DS11+ nanodrop spectrophotometer.

The gene for *Sm*TDK-PhoK was inadvertently cloned with the genetically encoded stop codon at the end of the DNA sequence which prevented its purification via HisTrap chromatography. The growth and expression were as described above. Following sonication and removal of cellular debris by centrifugation (see above) the supernatant was subjected to ammonium sulfate fractionation. The supernatant was brought to 35% saturation and incubated with stirring on ice for 20 min. Precipitated proteins were removed by centrifugation at 10k RPM for 10 min. The resultant supernatant was brought to 50% saturation with ammonium sulfate and stirred on ice for 20 min which was sufficient to precipitate *Sm*TDK-PhoK. The precipitated protein was recovered by centrifugation for 10 min at 10 kRPM. The protein was dissolved in 10 mL of 50 mM Hepes pH 8.0 and dialyzed against 3 changes of the same buffer. The protein was then passed over a 6 mL resource Q column from GE Healthcare on a GE Healthcare ACTA Prime Plus FPLC. The protein failed to bind to the column, but a significant proportion of the contaminating proteins did. The flow through was then concentrated to between 5 and 10 mL and subjected to size exclusion chromatography on the FPLC equipped with a High Prep 26/60 Sephacryl S200 HR column. Fractions with activity against *p*-nitrophenyl phosphate were subjected to SDS-PAGE, and pure fractions were concentrated to 2.8 mg/mL, flash frozen in liquid nitrogen and stored at −80 °C. All proteins were judged to be above 95% pure by SDS-PAGE.

### Kinetics by UV/Vis Spectroscopy

The kinetic constants for homologues with *p*-nitrophenyl phosphate (**1**) and phenyl phosphate (**2**) were determined by following the release of *p*-nitrophenol (*E*_400_ = 17,000 M^–1^ cm^–1^) or phenol (Δ*E*_275_ = 903 M^–1^ cm^–1^), respectively. Assays were 250 μL total volume with 50 mM Ches buffer (pH 9.0). Substrates were made as ∼10 mM stocks and serially diluted to achieve 32 evenly distributed concentrations between the high and low concentrations of the titrations (2 mM to 10 μM). Actual concentrations of stocks were determined by total hydrolysis of samples using concentrated enzyme. The high and low concentration for each titration is reported in Table S2. Assays were followed in a Molecular Devices Spectra Max ABS Plus 96-well plate reader. *p*-Nitrophenyl phosphate assays were conducted in Whatman Uniplate 96-well plates. Phenyl phosphate assays were conducted in Thermo Microtiter UV Flat Bottom 96-well plates. Reactions were initiated by addition of 10 μL appropriately diluted enzyme and followed for 10 min at the appropriate wavelength. Initial rates were fit to the Michaels Menton equation.

### Kinetics by ^31^P NMR

The hydrolysis of compounds **3**–**10** was followed in total hydrolysis assays monitored by ^31^P NMR as previously described.^32,33^ Assays were 1 mL total volume with 50 mM Hepes pH 8.0, 10% D_2_0 and 2.5 mM substrate. Reactions were initiated by addition of 10 μL of appropriately diluted enzyme. Enzymes were diluted to achieve 50% hydrolysis in between 1 and 3 h. During the course of the reaction the ^31^P NMR spectra was recorded every 15 min (128 scans, aqu = 3.5 s, d1 = 4 s) for 12 to 14 h. The total phosphorus signal for each spectra was integrated to calculate the fraction hydrolyzed at each time point. Each enzyme substrate combination was tested a minimum of twice with the initial test to determine the appropriate dilution of the enzyme followed by the total hydrolysis reaction. Plotting the fraction hydrolyzed as a function of time yielded first exponential curves that were fit to eq 1 to yield the exponential rate (*k*_obs_) as previously described.^33^

1 $$F=a\left(1-e^{-k_{obs}t}\right)$$

2 $$k_{cat}/K_{m}=\frac{k_{obs}}{\left[E\right]}$$

*F* is the fraction hydrolyzed, *a* is the magnitude of the exponential phase and *t* is time. The *k*_cat_/*K*_m_ for the enzyme is found by dividing the exponential rate by the enzyme concentration [*E*] as shown in eq 2. In some cases, the reactions demonstrated an initial linear phase followed by the expected first order exponential. In those cases, the data was reprocessed to only include the exponential phase.

## Supplementary Material


