# Supplementary material for: Broad Substrate Specificity and High Catalytic Activity of Sphingomonadaceae PhoK-Type Phosphatases Implicated in Flame-Retardant Degradation
Source: ACS Omega. 2025 Aug 29;10(36):41727–35. doi: 10.1021/acsomega.5c05616 (PMC12444516; doi:10.1021/acsomega.5c05616)
Supplement: Supplementary file 1 [file ao5c05616_si_001.pdf]

Supplementary Information

Broad Substrate Specificity and High Catalytic Activity of *Sphingomonadaceae* PhoK-type Phosphatases Implicated in Flame-retardant Degradation

Landry Freeman, Andrew Davis, Harley Gossen, Jake Estes and Andrew N. Bigley*

Department of Chemistry and Physics, Southwestern Oklahoma State University, Weatherford OK, USA, 73096

**Table S1**: Metal analysis by atomic absorption spectroscopy.	
**Enzyme**	**Zn per protein**
Sb-PhoK	1.8
Sy-PhoK	1.7
St-PhoK	1.8
*Sm*TDK1	1.8
No-PhoK	2.0
Na-PhoK	1.9
Sm-PhoK	1.8
*Sm*SRS-PhoK	1.7
Ng-PhoK	1.7

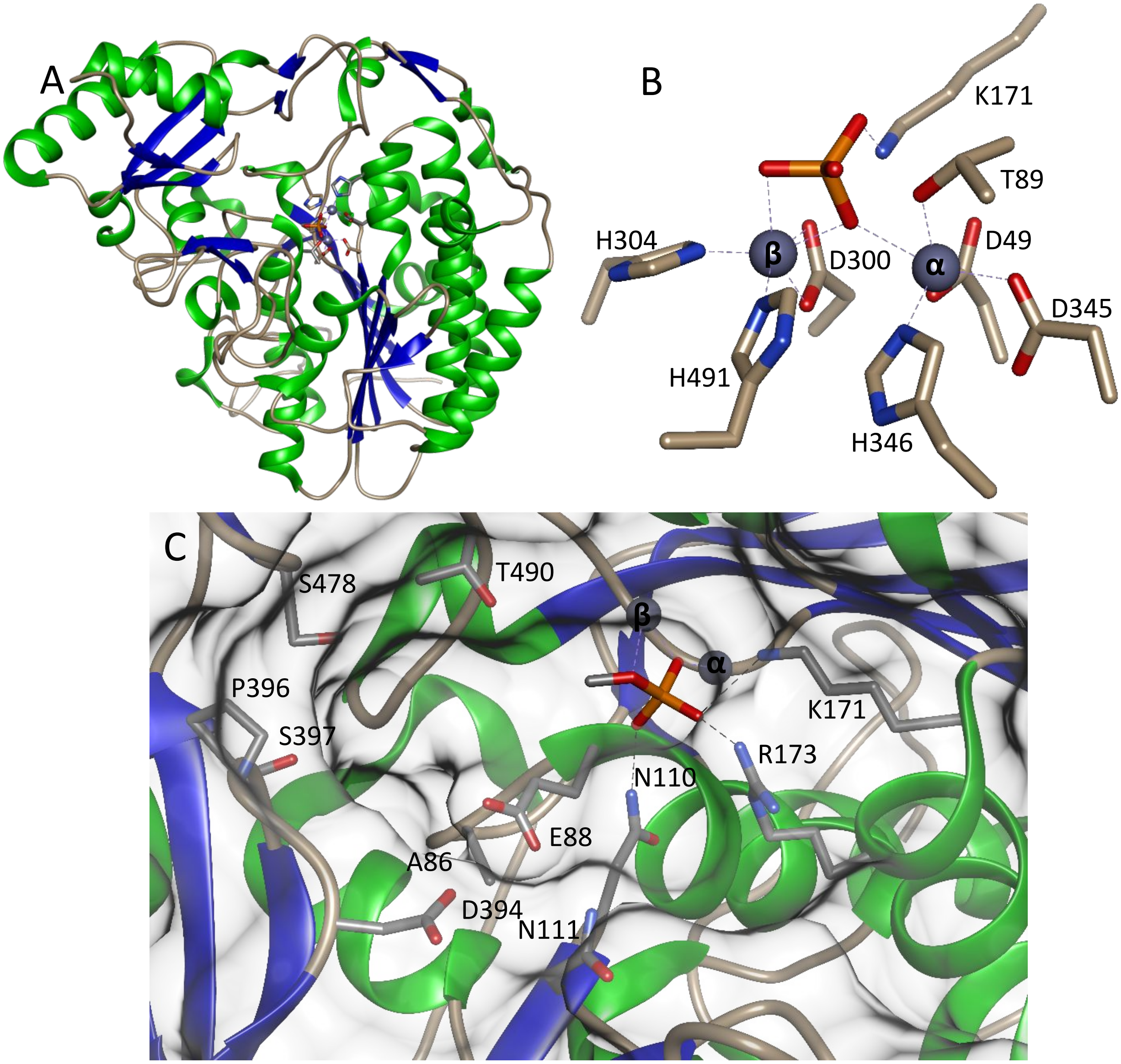

Figure S1: Crystal structure of *Sm*-PhoK. A shows overall structure with the binuclear metal center embedded at the C-terminal end of the central β-sheet. B shows the binuclear metal center with coordinating residues and phosphate bound in the active site. T89 is the proposed nucleophile in the catalytic mechanism. C shows the substrate binding residues K171, R173 and N110 as well as the ester group binding pocket lined by resides A86, E88, N111, D394, S397, P396, S478, and T490. Panels A and B are from pdb:5xwk, and panel C is from pdb:3q3q.

```
Sm-PhoK      20-------------------QSPAPAAAPAPAARSIAATPPKLIVAISVDQFSADLFSEYRQYYTGGLKRLTSEGAVFPRGYQSHAATETCPGHSTILTGSRPSRTGIIANNWFDLDAKREDKNLYCAEDE 130
Sb-PhoK      20MFKRVAAALLLASAMPVMAQTPAPAPV---AATVAPVAAPKLIVAISIDQFSADLFSEYRQYYSAGLKRLT-QGAVFPRGYQSHAATETCPGHSTILTGSRPARTGIIANSWFDLGTGREDKAIYCAEDE 145
SmTDK1-PhoK  21-----------------------QQAAP---APALPASPPKLVVVVSVDQFSADLFAEYRSQFTSGFARLM-SGAVFPAGYQSHAATETCPGHSTILTGDHPSRTGIIANDWTDFAAPRADKTVYCAEDE 123
Sp-PhoK      29-----------------------QDSAPPVAKVTAATPPPKLIVAISVDQFSADLFAEYRSHYTGGLARLA-SGVVFPAGYQSHGATETCPGHATILTGNHPAHTGIIANNYFDLSVARSDKRVYCAEDE 134
Sy-PhoK      19-------------------QSPAPAAAPAPAASSIAATPPKLIVAISVDQFSADLFSEYRQYYTGGLKRLTSEGAVFPRGYQSHAATETCPGHSTILTGSRPSRTGIIANNWFDLDAKREDKNLYCAEDE 129
Na-PhoK      26------------------QEAPPPPPSLPIVSQQVPQNAPRLVVAISIDQFSSDIFAQYRQRFTGGLARLT-TGAVFPAGYQSHAATETCPGHSTILTGNRPAHTGIIANSWIDQSVAVGPKQVYCAEDT 136
No-PhoK      25----------------RPAKAPKPAPAPVEAPAPAPKGPPRLLVAIAVDQFSADLFAQNRQTFTGGLARLE-RGMVFPAGYQSHAATETCPGHSTILTGNRPAHTGIIANNWIDLSAARDNKVIYCAEDE 137
SmSRS2-PhoK  20----------------------------------APPPKPKLIVAISVDQYSSEVFKRYRPTYTAGLKRLA-NGVAYPTGYQSHAATETCPGHSTILTGRHPAGTGIVANSWIDAKTG---MSVYCVSVP 112
Ng-Phok      22---------------QEAAPSSAPAPAPAPAAEAAPRTPPKLIVAISVDQYSSDLFQQYRERYSKGLNRLL-TGAVFPSGFQSHAATETCPGHSTLLTGVHPNRTGIPANSWFEPGIGREDKEVYCSEDE 135
                        .....  ..  .: ..*:*:*::::**:*:::*..:*..::.*:.**. .*:::*.*:***:*******:*:***.:*..***:**.:.:.  .....::**::::

Sm-PhoK        SQPGSSS--DKYEASPLHLKVPTLGGRMKAANPATRVVSVAGKDRAAIMMGGATADQVWWLGGPQGYVS--YKGVAPTPLVTQVNQAFAQRLAQPNPGFELPA------QCVSKDFPVQA---------- 240
Sb-PhoK        SAPGSNS--DAYVASPVHLKVPTLGGRMKAANPATRVVSVAGKDRAAIMMGGPTADQVWWLGGAQGYVS--YKGVPTPPLVAKVNQVFAQRLAQPNPGFELPA------QCAAKDFPVQA---------- 255
SmTDK1-PhoK    TVPGTDS--NHYTVSDKHLKVPTLGEWMKQADPQSRVVSVAGKDRAAVMMGGHKVDELWWWDG-KAFVS--YAGRTAPDAVTKINAATSARIAEAQPALDLPA------FCKARSRAIPV---------- 232
Sp-PhoK        TVPDTSSGGGKYAASVNHLMVPTLGDLMKARDPKAQIVSVAGKDRAAIMMGGRKADELMWLFP-GGLTS--YRGTTLSPVAQQASSAIAAAIGQARPAMDLPA------DCASHDIAIPI---------- 244
Sy-PhoK        SQPGSSS--DKYEASPLHLKVPTLGGRMKAANPATRVVSVAGKDRAAIMMGGATADQVWWLGGPQGYVS--YKGVAPTPLVTQVNQAFAQRLAQPNPGFELPA------QCVSKDFPVQA---------- 225
Na-PhoK        GKRAAGS--KDYVASPIHLLVPTLGEWMKRANPAARNIAVSGKDRGALMMGGHDTDQVYWWKG-KGFVT--LAGREPGPTAIAQNVEIARTLAKGAPAFPLPA------YCGRNDRAVTA---------- 244
No-PhoK        TRTAANP--KDYVASVGHLMVPTLGERMKQANPASRNVAVSGKDRAALMMGGHVIDQVYWWKG-NAFTT--LEGRKLGPTAVAENAAVAKVLAAGAPAFDLPA------WCVAKDRAITA---------- 245
SmSRS2-PhoK    GVAE------KSARGPQNMRVTTLGDWIKAAQPRARSFAVAGKDRAAITMAGKNADGVYWWVDGTGFTTSSYAGPATPRI-VKPAEAFNAKLMSAWKATPPQLWPAASPTCAALSKRQQFGQLEVSGAMP 234
Ng-Phok        TNPASNS--QDSVISPMHLKVPTLGDLLKAKNPATLNVAVSAKDRAVVMMSGHTSDAAYWIKG-KGFVT--YDDRKLSAAAVAQNAAITKAVEAGDGDLPAPG------WCAPYDHAVNL---------- 243
                . ... .   .:..:. ::.*:***..:*.::*..:.::*::***:::*:*...*.:.*. : .::::  :.:........ :.:...:......::       *...: ....

Sm-PhoK        ----GNRTVGTGRFARDAGDYKGFRISPEQDAMTLAFAAAAIENMQLGKQAQTDIISIGLSATDYVGHTFGTEGTESCIQVDRLDTELGAFFDKLDKDGIDYVVVLTADHGGHDLPERHRMNAMPMEQR  365
Sb-PhoK        ----GNRTVGTGRFARDAGDYNGFRISPEQDAMTLAFAAAAIETMGLGKQTQTDIISIGLSATDYIGHTYGTEGTESCIQVDRLDQELGAFFDRLDKDGIDYVVVLTADHGGHDLPERHRLNAMPMESR  380
SmTDK1-PhoK    ---SATKTVGDGRFARAAGDAKAFRISPEFDDAVLALGAKLATDMKLGQQGHTDLLILGASATDYVGHTYGTEGSEMCLQLLSLDQTLGRLFDALDRTNVDYQVVLTADHGGHDLPERHREHGAPTEQR  358
Sp-PhoK        ---DKGGTVGTGRFQRDGGDFRHFMASPEADGAVLAAGAALRAARKLGEGTTTDLLILGLSATDYIGHSTGTEGAEMCIQMHALDQQLGDFFARLDATGIDYAVVLTADHGGHDLPERNRQNAWPAAER  371
Sy-PhoK        ----GNRTVGTGRFARDAGDYKGFRISPEQDAMTLAFAAAAIESMQLGKQAQTDIISIGLSATDYVGHTFGTEGTESCIQVDRLDTELGAFFDKLDKDGIDYVVVLTADHGGHDLPERHRMNAMPMEQR  364
Na-PhoK        ----GEVTVGTNHFALKAGDADGFRISPRLDRATLDLAVKLVDEQKLGRGAVPDLLAVSLSATDYVGHATGTEGAEMCIQLAQLDLALGDFLASLDKRGIDYAVVLTADHGGFDIPERLHEQALEKSAR  370
No-PhoK        ----GTATVGTGRFPLAKDDATGFRVSPRLDRATLDLATKLVDEQKLGQGSAPDMLAVSLSATDYIGHGTGTQGAEMCIQVHQLDLALGDFFAGLDKRGIDYAVVLTADHGGFDMPERQDEQAMPQVTR  371
SmSRS2-PhoK    PDMAKGVTE-GDEFLSSKRFNDAMHASPTFDAAVADFAIDLIEREKLGRGSATDVLAVGLSATDYIGHRLGNGGPEMCVQQAALDATIGRLIDRIQALRIPVMVMLTADHGATDASERHFDEGGHG-YR  361
Ng-Phok        ----GSFSIGTFRFPIKPGDYKSYLNGPRVDDVTTDMAIRLLDEMPLGKDDVPDVLSVSYSANDKVGHAFGTEGVEMCIQQNVLDATIGRLLAALDARGIDYAVVLSADHGGIDAPERLKLQGVPEASR  369
                   .. ::::.:*....::...:..:*. *..::..:..:.....**....:*.:.:::**:*::**..*::*.*:*:*..**..:*.::..::. :: ::.*:*:****:.*.:**.. .: :   *

Sm-PhoK        VDMALTPKALNATIAEKAGLPGK--KVIWSDGPSGDIYYDKGLTAAQRARVETEALKYLRAHPQVQTVFTKAEIAATPSPSGPP-ESWSLIQEARASFYPSRSGDLLLLLKPRVMSIPEQAVMGSVATH  491
Sb-PhoK        VDMALTPKALSAAVAEKAGLPGR--KVIWGDGPAGDLYFDKGLTAAQRARVEAEALKLLRAHPQVETVFTKAEIAATPSPSGPP-ESWSLIQEARASFDSERSGDLLLLLKPRVMSIPERAVMGAVATH  506
SmTDK1-PhoK    ITADVTTTNISRALAGKLSLTG---PVLVG-GPGGDVYVTPSVPQAKRAAVLAEAAKLYAANPQVQAVFTRAQIEATPIVHTPP-ETWSLLERARASYDPQRSGDLIVALKPRVTPILDP-GVGNVATH  481
Sp-PhoK        VDAALDPEAMGKAVAEKLGLPQ---PLLYGDGPFGDMYLSKALTPAQRKAALAELVARYRAHRQVEAVVTGEELANHPISKRSP-DVWTLMDKLRASYNAQRSGDFIVVLKPRVTPIPES-GLGYVATH  495
Sy-PhoK        VDMALTPKALNATIAEKAGLPGK--KVIWSDGPSGDIYYDKGLTAAQRARVETEALKYLRAHPQVQTVFTKAEIAATPSPSGPP-ESWSLIQEARASFYPSRSGDLLLLLKPRVMSIPEQAVMGSVATH  492
Na-PhoK        VGPEVSAEALSEALGKRYGLAPK--GLILADGPAGDYWLRKDLDEGLRGRIVDDAKAILMTSPYVEKVLSAAEIAATPMPSGSP-ETWTLAERARASYNPLHSGDFVVMLKRSVVAIPTP-RAGYVATH  495
No-PhoK        VDAALQPDAIGKAVAEKLGLDPR--GLIRSDGPAGDIWLRRDLTAEQKARVTDETKAMFAAHPQVAAVFTAAEIAATPMPSGSP-ETWTLIQRARASYYAPRSGDLLVLLKRGVVPIAKP-GPGYVATH  496
SmSRS2-PhoK    IDDKSLMNRLNDALEAELGIVWT--PL--NTEDPQQLYVVVDSEEAPRERILHSAVAWLKAQPEVRAVFTRAEVEAATVPPGTPADKLTVLQRFRESYDAERSGDIAVAYAERASYGVPNAVGETVAGH  486
Ng-Phok        LDPALTDKGLSALVSKATGIVPKDGPLLYGAGASGDIWLNAGLTAAQRKKVIATLMTQLKANPQIAGAYTADELAKVKVPAGHP-QDWTLIERVAASFVPGRSGDVLMMTKRAVVPGVAP-RPGGASTH  496
               ::.......:..::....::..   .:. ..::.::.:. ..:..:.:.::...... :.:.::..::.:::::.:.:.:.* :.::.::.::*:.. :***..:.::.:...... . : :::*

Sm-PhoK        GSPWDTDRRVPILFWRKGMQHFEQPLGVETVDILPSLAALIKLPVPKDQIDGRCLDLVAGKDDSCAGQ-   559
Sb-PhoK        GSPWDTDRRVPILFWRKDMRHFEQPLGVETVDIMPSLAALIGLPVAKGEIDGRCLDLIAGAGDSCAPE-   574
SmTDK1-PhoK    GSFWDYDRRVPILFWRKGMTGFEQPLSVETVDIAPTLAALIKVPVP-VEIDGRCLDIDAGAGDSCK---   547
Sp-PhoK        GSVWDYDRRVPILFWRKGLTAFEQPNAVMTVDIMPTLAALIDLPVDAAKIDGRCLDLLSGPESSCRP--   563
Sy-PhoK        GSPWDTDRRVPILFWRKGMQHFEQPLGVETVDILPSLAALIKLPVPKDQIDGRCLDLVAGKDDSCAGQ-   559
Na-PhoK        GSPWDYDRRVPIMFWRKGMNGFEQPSAVETVDIAPSLAALIGLKIPQGAFDGRCLDLDGGPANTCGAGK   565
No-PhoK        GSPWDYDRRVPILFWRKGMTPFEQPGPVETVDIAPTLAGLIALKAPEGAFDGRCLDLDAGPGDTCGANR   565
SmSRS2-PhoK    GTPWDYDRQIPILFWWPGAPAETRDQPIETVDIAPTLASVAGITPP-VPVDGDCLDLGGN----CRK--   549
Ng-Phok        GSPWDYDRRVPMLFWRKGLTTMEQPQPVETVDIAPTLAALVGLSEPAGTWDGRCLDIYAGEADSCSR--   564
               *::**.**::*::**::::...::: .::****.*:**:::.:..: ...**:***: ::   ..*.
```

Figure S2: Sequence alignment of PhoK homologs with *Sm*-PhoK which has a known crystal structure (pdb:3Q3Q). Residues highlighted in red are metal ligands. Residues highlighted in yellow H-bond to substrate, and residues highlighted in blue line the substrate binding pocket.

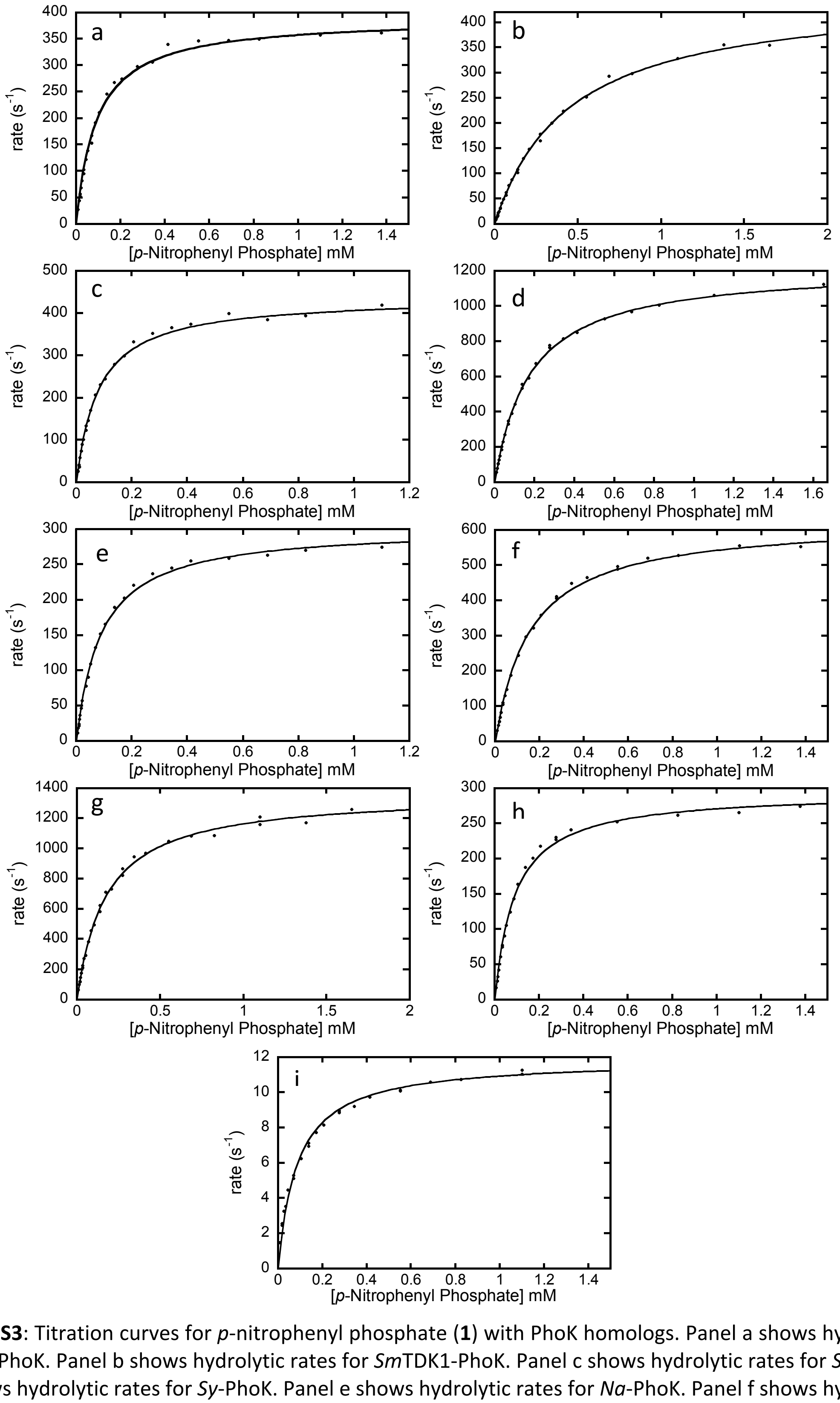

Figure S3: Titration curves for *p*-nitrophenyl phosphate (**1**) with PhoK homologs. Panel a shows hydrolytic rates for *Sb*-PhoK. Panel b shows hydrolytic rates for *Sm*TDK1-PhoK. Panel c shows hydrolytic rates for *St*-PhoK. Panel d shows hydrolytic rates for *Sy*-PhoK. Panel e shows hydrolytic rates for *Na*-PhoK. Panel f shows hydrolytic rates for *No*-PhoK. Panel g shows hydrolytic rates for *Sm*-PhoK. Panel h shows hydrolytic rates for *Ng*-PhoK, and panel i shows hydrolytic rates for *Sm*SRS-PhoK.

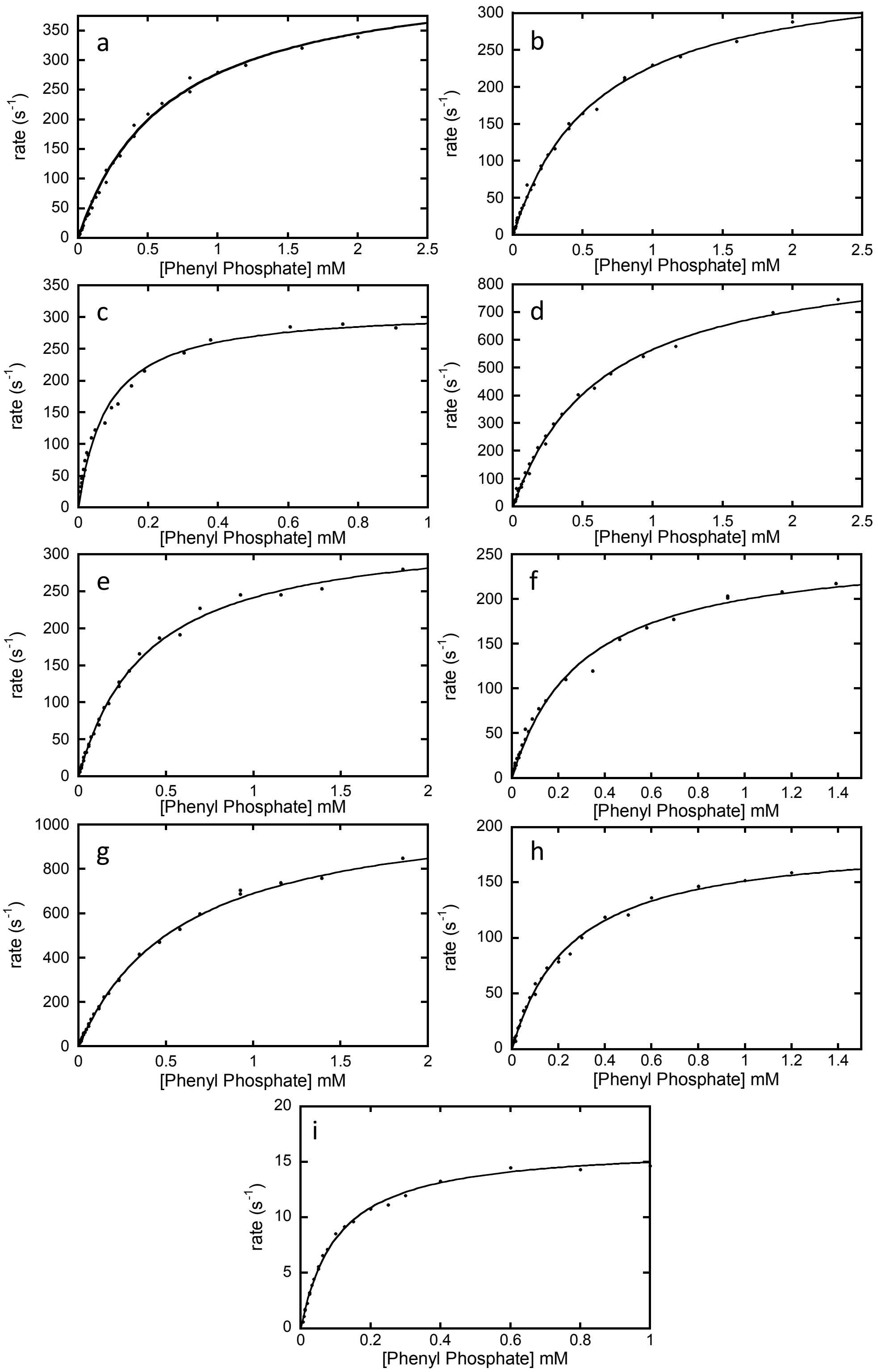

Figure S4: Titration curves for phenyl phosphate (**2**) with PhoK homologs. Panel a shows hydrolytic rates for *Sb*-PhoK. Panel b shows hydrolytic rates for *Sm*TDK-PhoK. Panel c shows hydrolytic rates for *St*-PhoK. Panel d shows hydrolytic rates for *Sy*-PhoK. Panel e shows hydrolytic rates for *Na*-PhoK. Panel f shows hydrolytic rates for *No*-PhoK. Panel g shows hydrolytic rates for *Sm*-PhoK. Panel h shows hydrolytic rates for Ng-PhoK, and panel i shows hydrolytic rates for *Sm*SRS-PhoK.

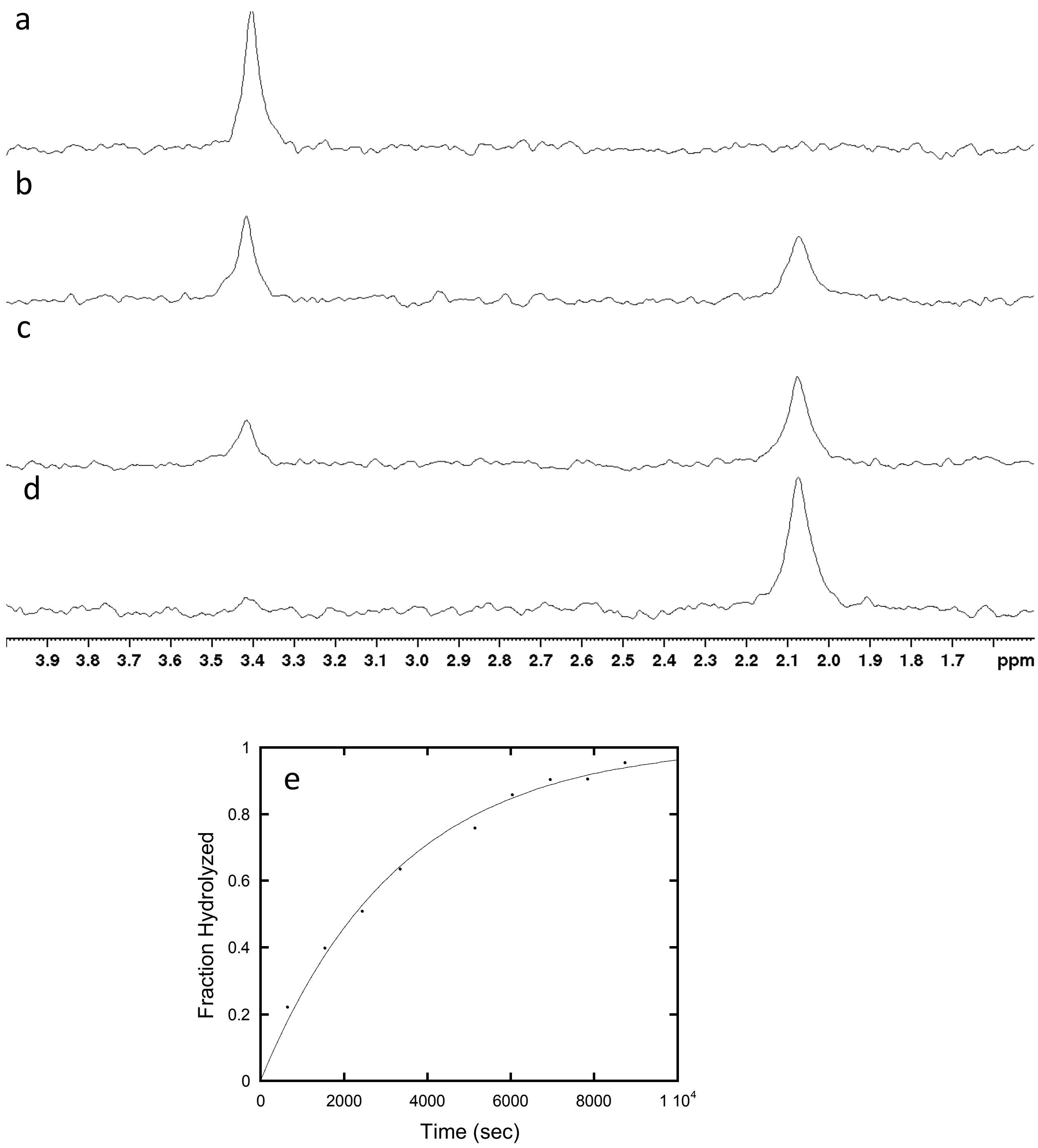

Figure S5: Hydrolysis of 2.5 mM 2-chloroethyl phosphate (**3**) by 8.1 nM *Sb*-PhoK followed by ^{31}P NMR. Panel a shows the ^{31}P NMR spectra of 2-chloroethyl phosphate (**3**) with resonance at 3.41 ppm before addition of enzyme. Panel b shows appearance of the phosphate product at 2.05 ppm 41 min after addition of enzyme. Panel c shows reaction after 71 min, and panel d shows product after 146 min. Panel e shows exponential curve fit to NMR data.

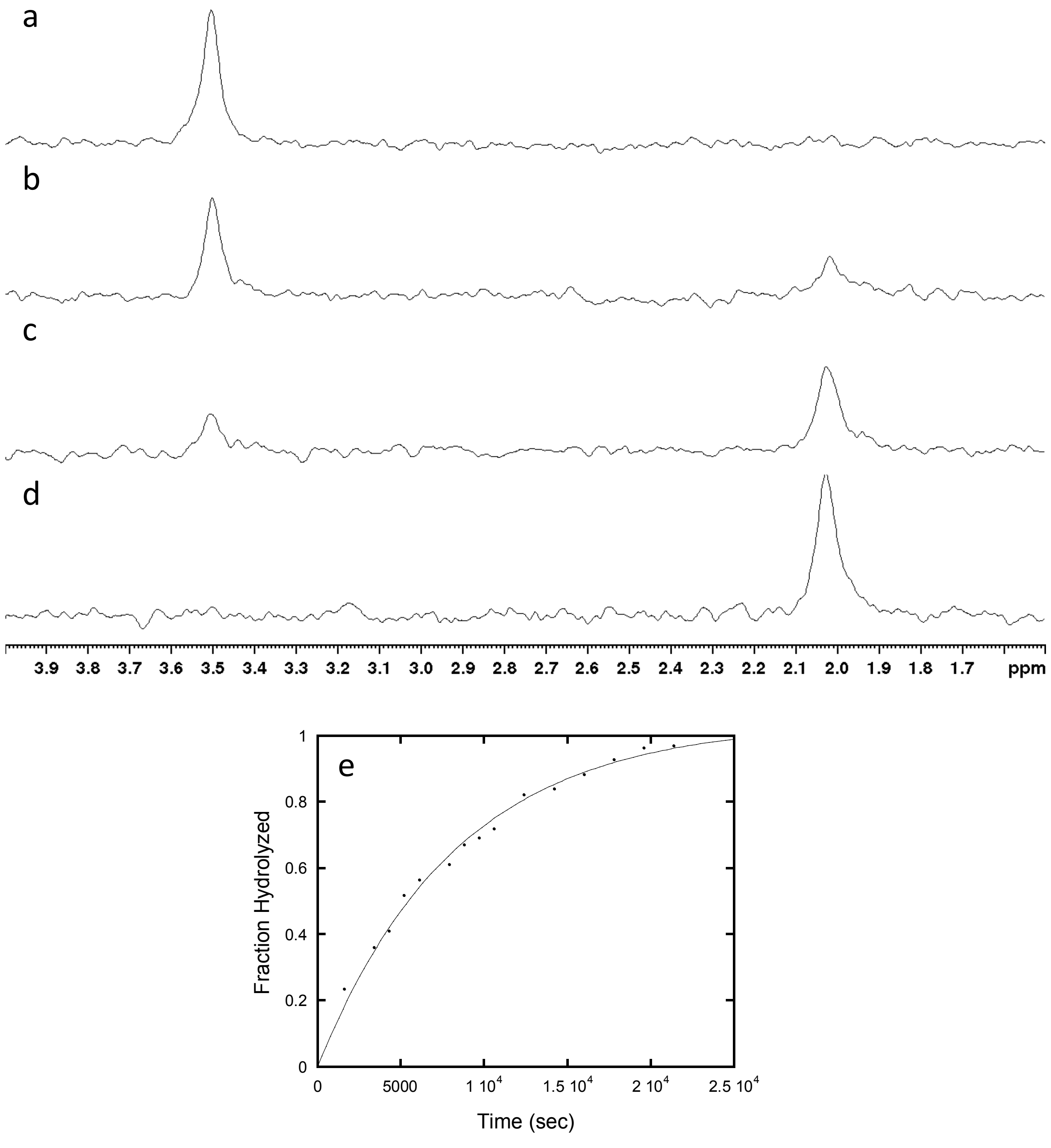

Figure S6: Hydrolysis of 2.5 mM 2-butoxethyl phosphate (**5**) by 4.1 nM *Sb*-PhoK followed by ^{31}P NMR. Panel a shows the ^{31}P NMR spectra 2-butoxethyl phosphate (**5**) with resonance at 3.51 ppm before addition of enzyme. Panel b shows appearance of the phosphate product at 2.03 ppm 57 min after addition of enzyme. Panel c shows reaction after 162 min, and panel d shows product after 342 min. Panel e shows exponential curve fit to NMR data.

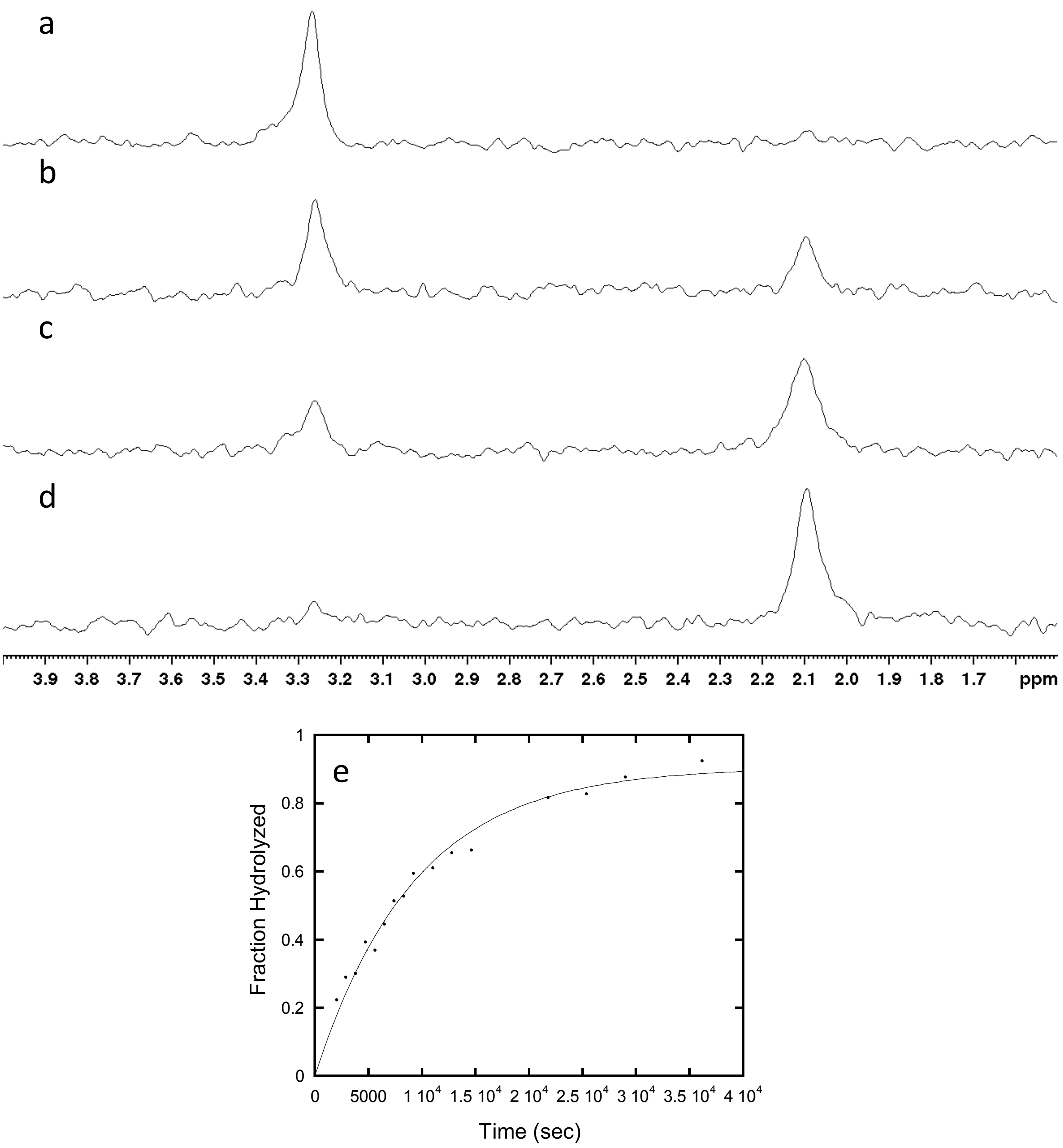

Figure S7: Hydrolysis of 2.5 mM butyl phosphate (**6**) by 8.1 nM *Sb*-PhoK followed by ^{31}P NMR. Panel a shows the ^{31}P NMR spectra butyl phosphate (**6**) with resonance at 3.26 ppm before addition of enzyme. Panel b shows appearance of the phosphate product at 2.09 ppm 93 min after addition of enzyme. Panel c shows reaction after 303 min, and panel d shows product after 723 min. Panel e shows exponential curve fit to NMR data.

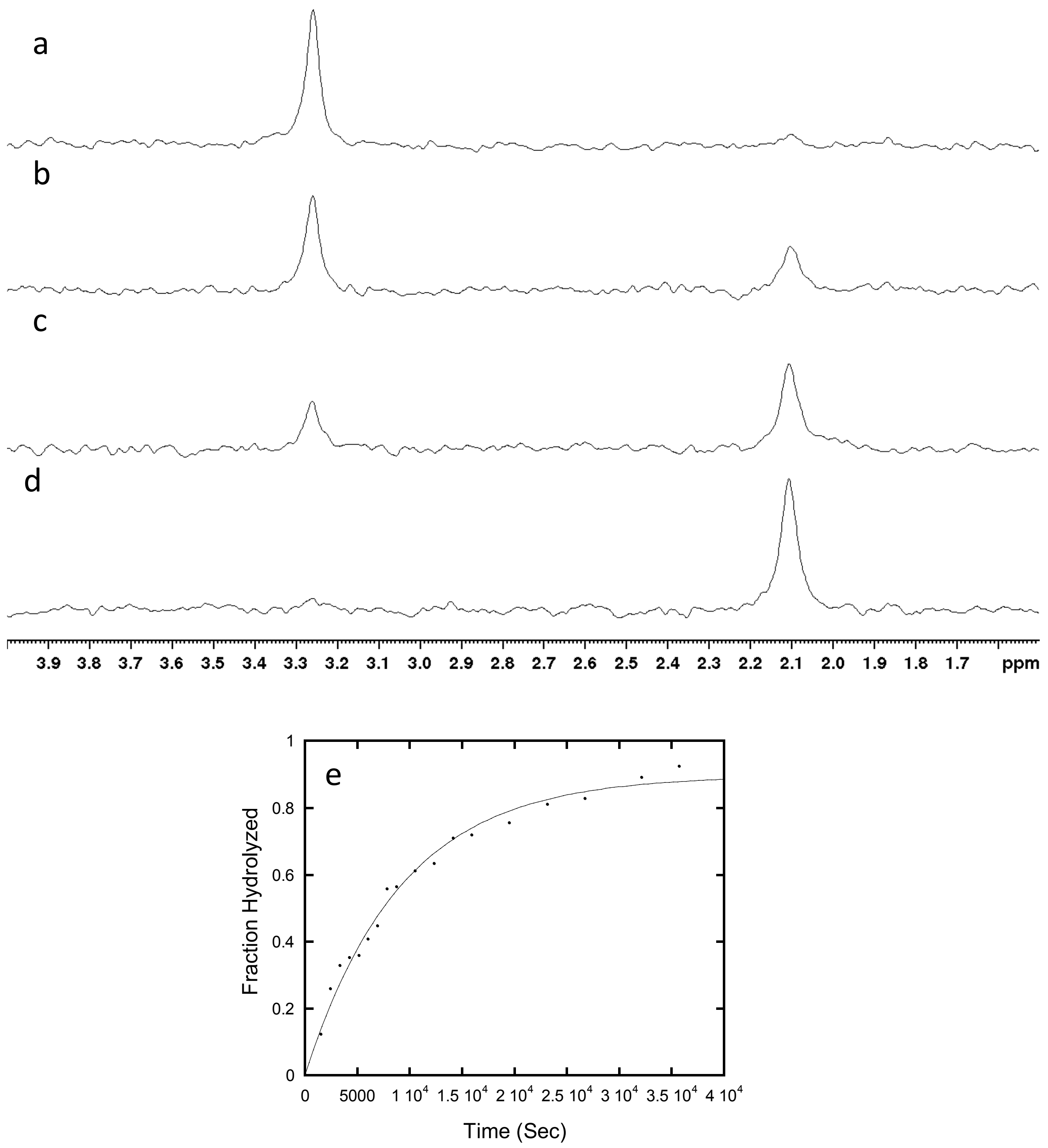

Figure S8: Hydrolysis of 2.5 mM ethyl phosphate (**7**) by 4.05 nM *Sb*-PhoK followed by ^{31}P NMR. Panel a shows the ^{31}P NMR spectra ethyl phosphate (**7**) with resonance at 3.26 ppm before addition of enzyme. Panel b shows appearance of the phosphate product at 2.10 ppm 70 min after addition of enzyme. Panel c shows reaction after 235 min, and panel d shows product after 715 min. Panel e shows exponential curve fit to NMR data.

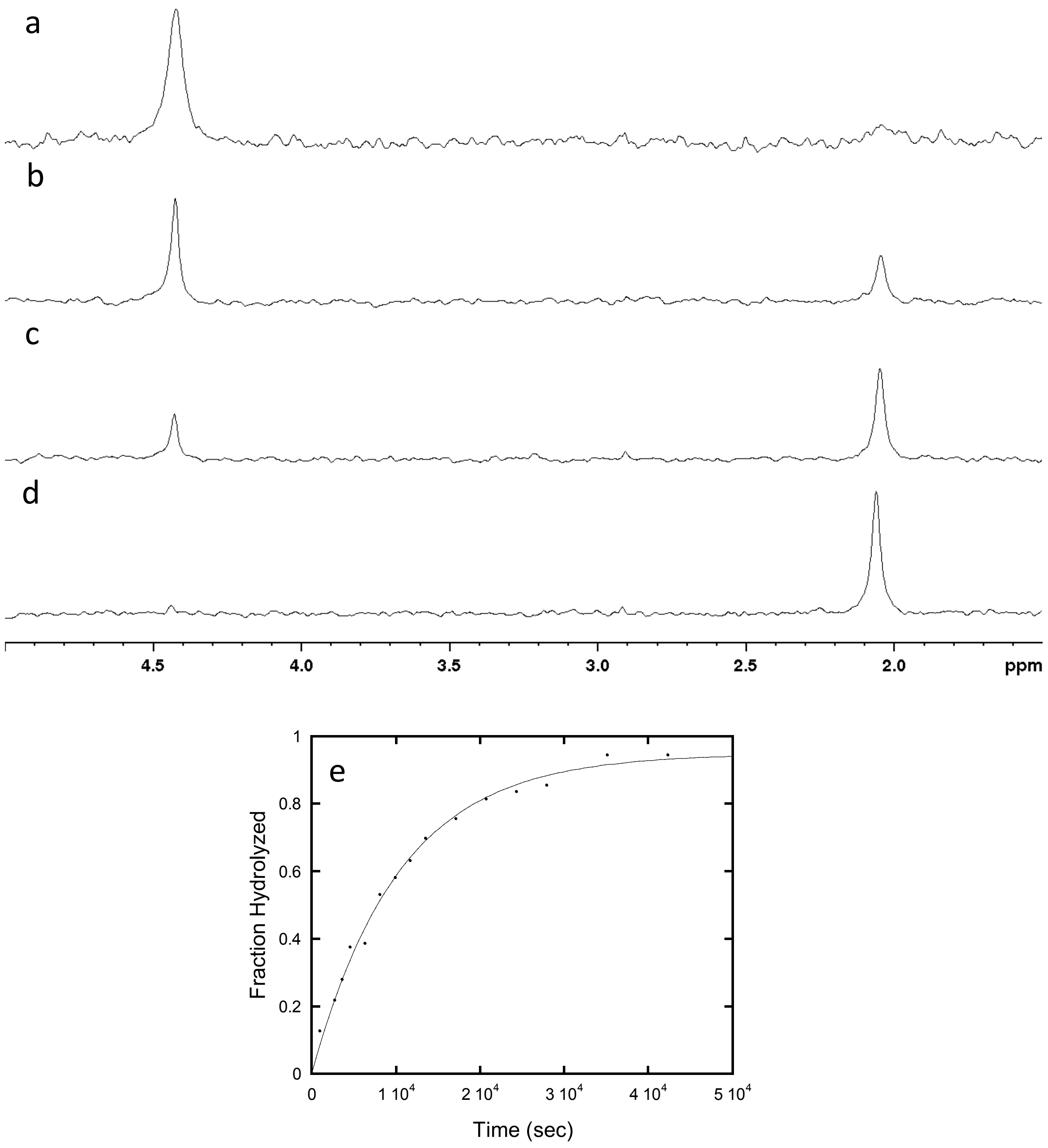

Figure S9: Hydrolysis of 1.25 mM methyl phosphate (**8**) by 8.2 nM *Sb*-PhoK followed by ^{31}P NMR. Panel a shows the ^{31}P NMR spectra methyl phosphate (**8**) with resonance at 4.42 ppm before addition of enzyme. Panel b shows appearance of the phosphate product at 2.05 ppm 70 min after addition of enzyme. Panel c shows reaction after 235 min, and panel d shows product after 715 min. Panel e shows exponential curve fit to NMR data.

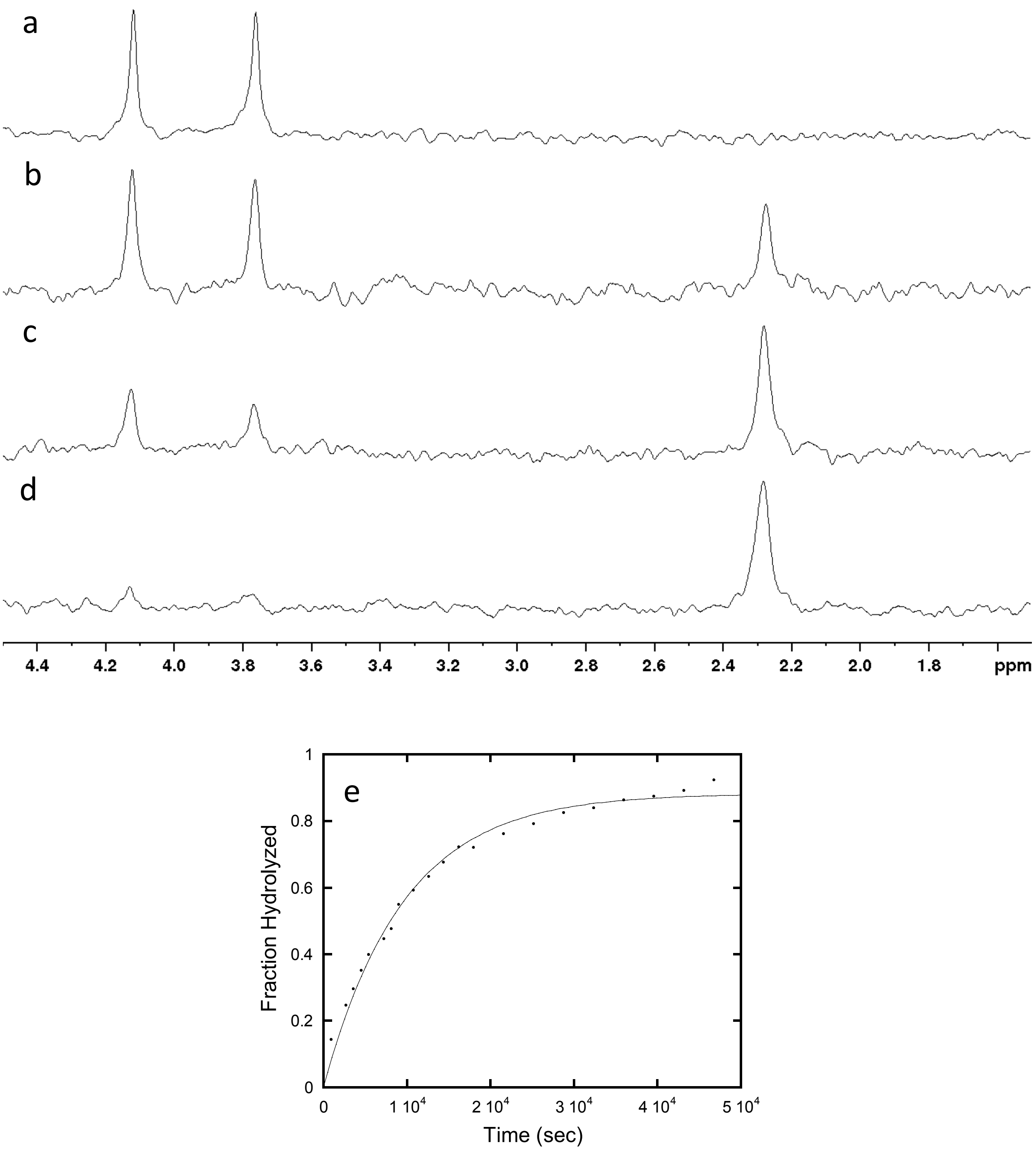

Figure S10: Hydrolysis of 2.5 mM glycerol phosphate (**9**) by 4.1 nM *Sb*-PhoK followed by ^{31}P NMR. Panel a shows the ^{31}P NMR spectra glycerol phosphate (**9**) before addition of enzyme. Resonance of the α-isomer is at 4.12 ppm, and resonance from the β-isomer is at 3.77 ppm.[2] Panel b shows appearance of the phosphate product at 2.27 ppm 59 min after addition of enzyme. Panel c shows reaction after 224 min, and panel d shows product after 780 min. Panel e shows exponential curve fit to NMR data.

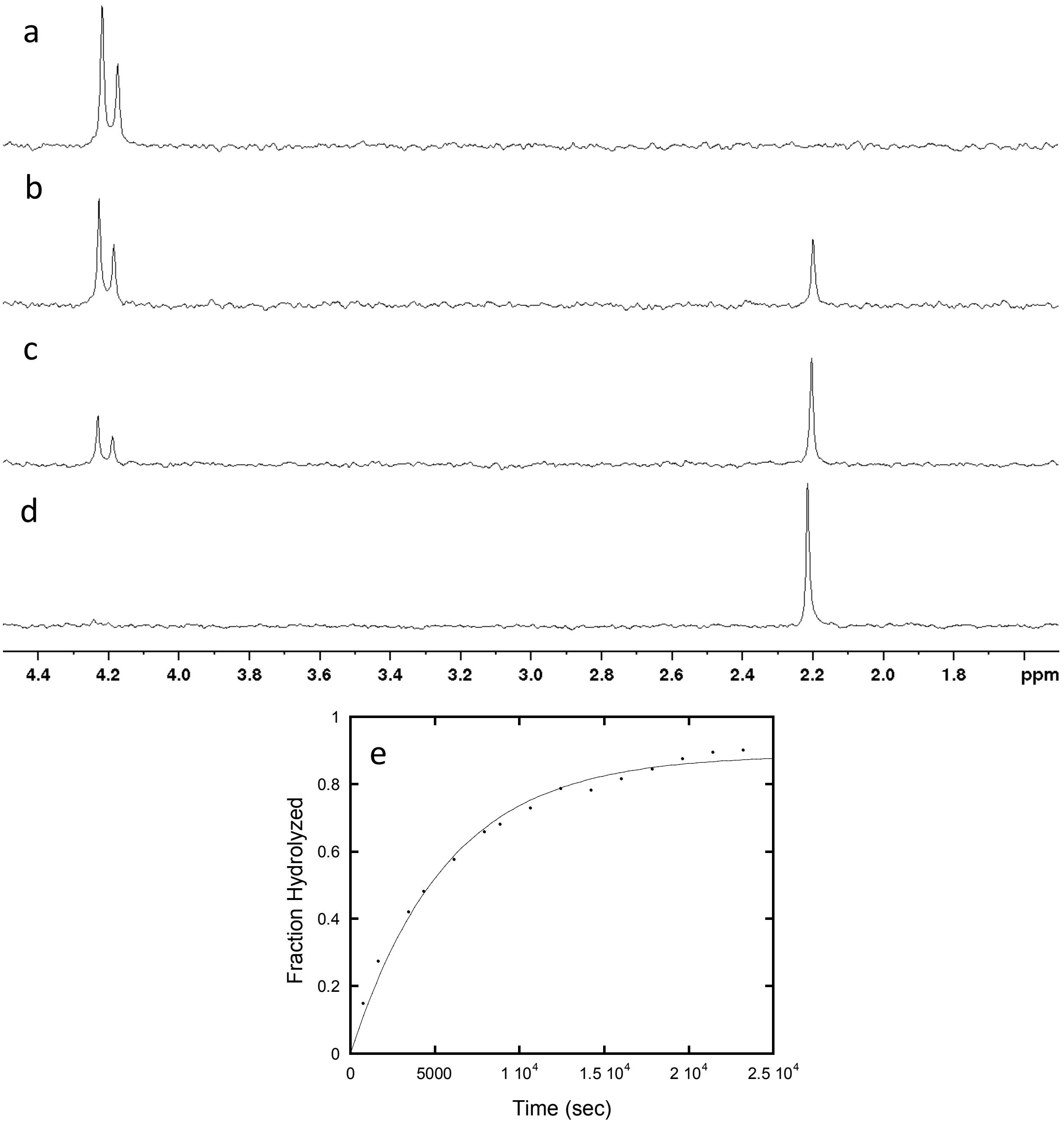

Figure S11: Hydrolysis of 2.5 mM glucose-6-phosphate (**10**) by 8.2 nM *Sb*-PhoK followed by ^{31}P NMR. Panel a shows the ^{31}P NMR spectra glucose-6-phosphate (**10**) before addition of enzyme. The α-isomer resonates at 4.17 ppm, and the β-isomer resonates at 4.21 ppm.[1] Panel b shows appearance of the phosphate product at 2.20 ppm 28 min after addition of enzyme. Panel c shows reaction after 117 min, and panel d shows product after 716 min. Panel e shows exponential curve fit to NMR data.

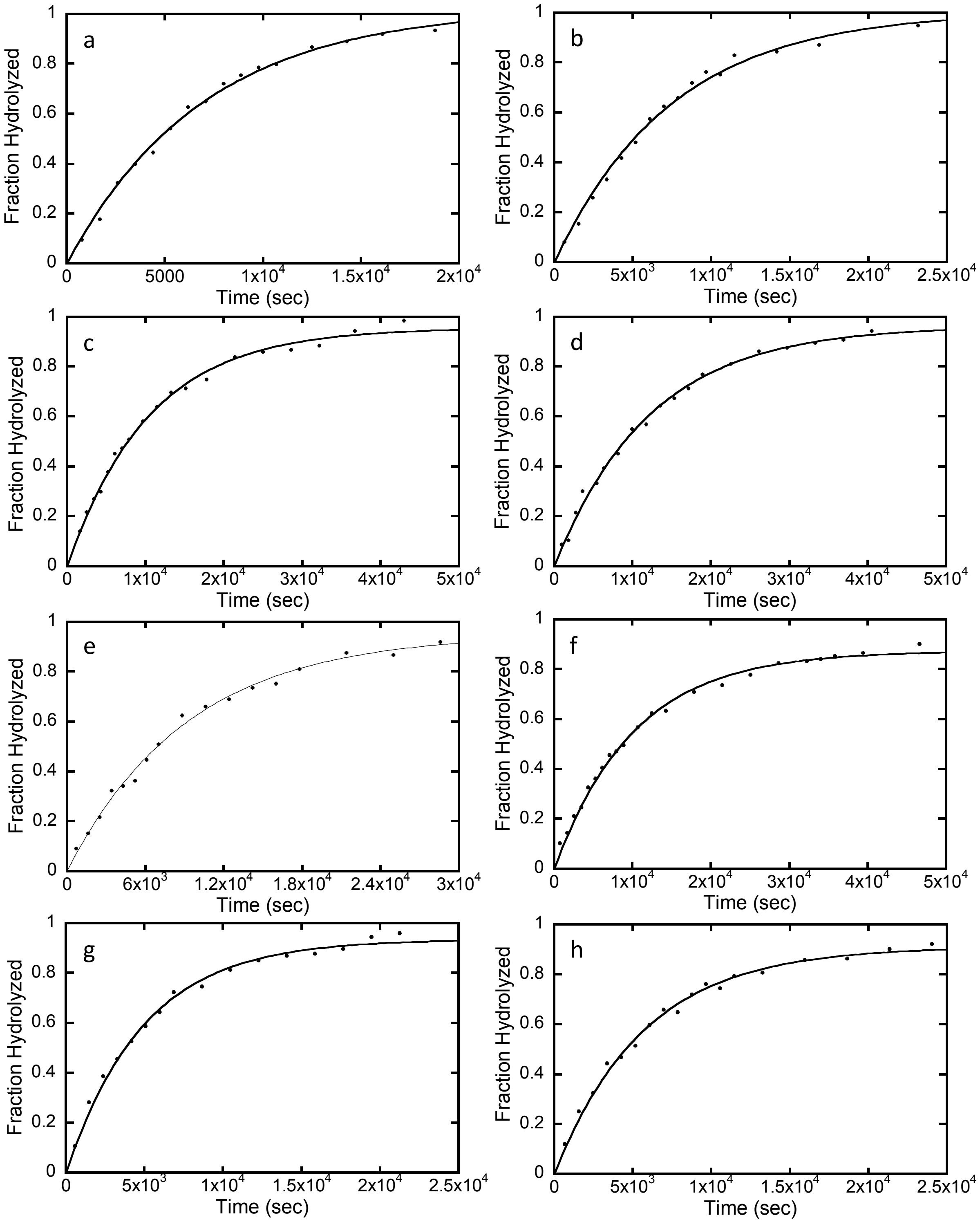

Figure S12: Time courses for the hydrolysis of compounds **3**-**10** by *Sm*TDK1-PhoK followed by ^{31}P NMR. Panel a shows hydrolysis of 2-chloroethyl phosphate (**3**). Panel b shows hydrolysis of 1,3-dichloroisopropyl phosphate (**4**). Panel c shows hydrolysis of 2-butoxyethyl phosphate (**5**). Panel d shows hydrolysis of butyl phosphate (**6**). Panel e shows hydrolysis of ethyl phosphate (**7**). Panel f shows hydrolysis of methyl phosphate. Panel g shows hydrolysis of glycerol phosphate (**9**, mixed isomers). Panel h shows hydrolysis of glucose-6-phsophate (**10**). All data are fit to equation 1 which yields the k_{cat}/K_m for the reaction.

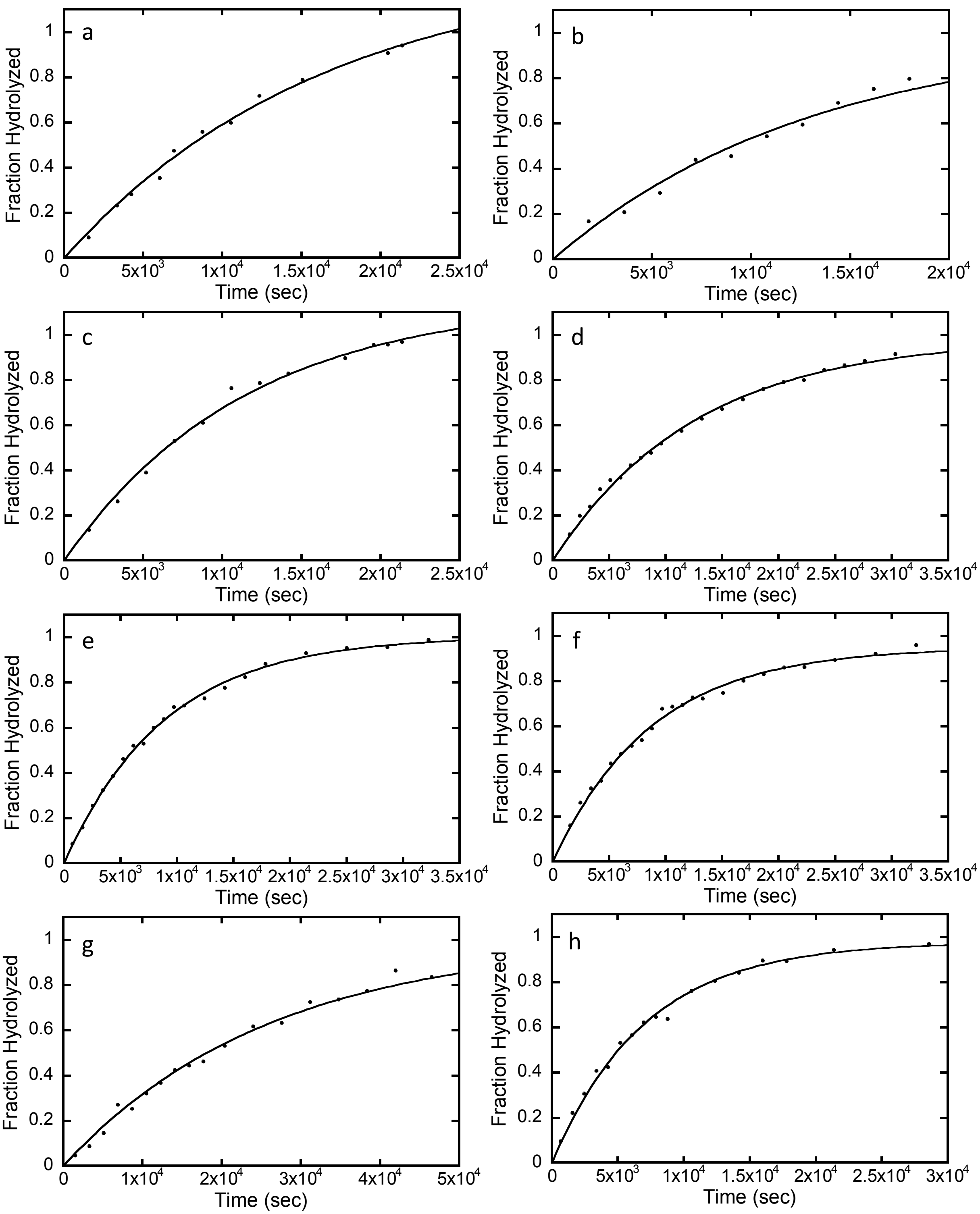

Figure S13: Time courses for the hydrolysis of compounds **3**-**10** by *St*-PhoK followed by ^{31}P NMR. Panel a shows hydrolysis of 2-chloroethyl phosphate (**3**). Panel b shows hydrolysis of 1,3-dichloroisopropyl phosphate (**4**). Panel c shows hydrolysis of 2-butoxyethyl phosphate (**5**). Panel d shows hydrolysis of butyl phosphate (**6**). Panel e shows hydrolysis of ethyl phosphate (**7**). Panel f shows hydrolysis of methyl phosphate. Panel g shows hydrolysis of glycerol phosphate (**9**, mixed isomers). Panel h shows hydrolysis of glucose-6-phsophate (**10**). All data are fit to equation 1 which yields the k_{cat}/K_m for the reaction.

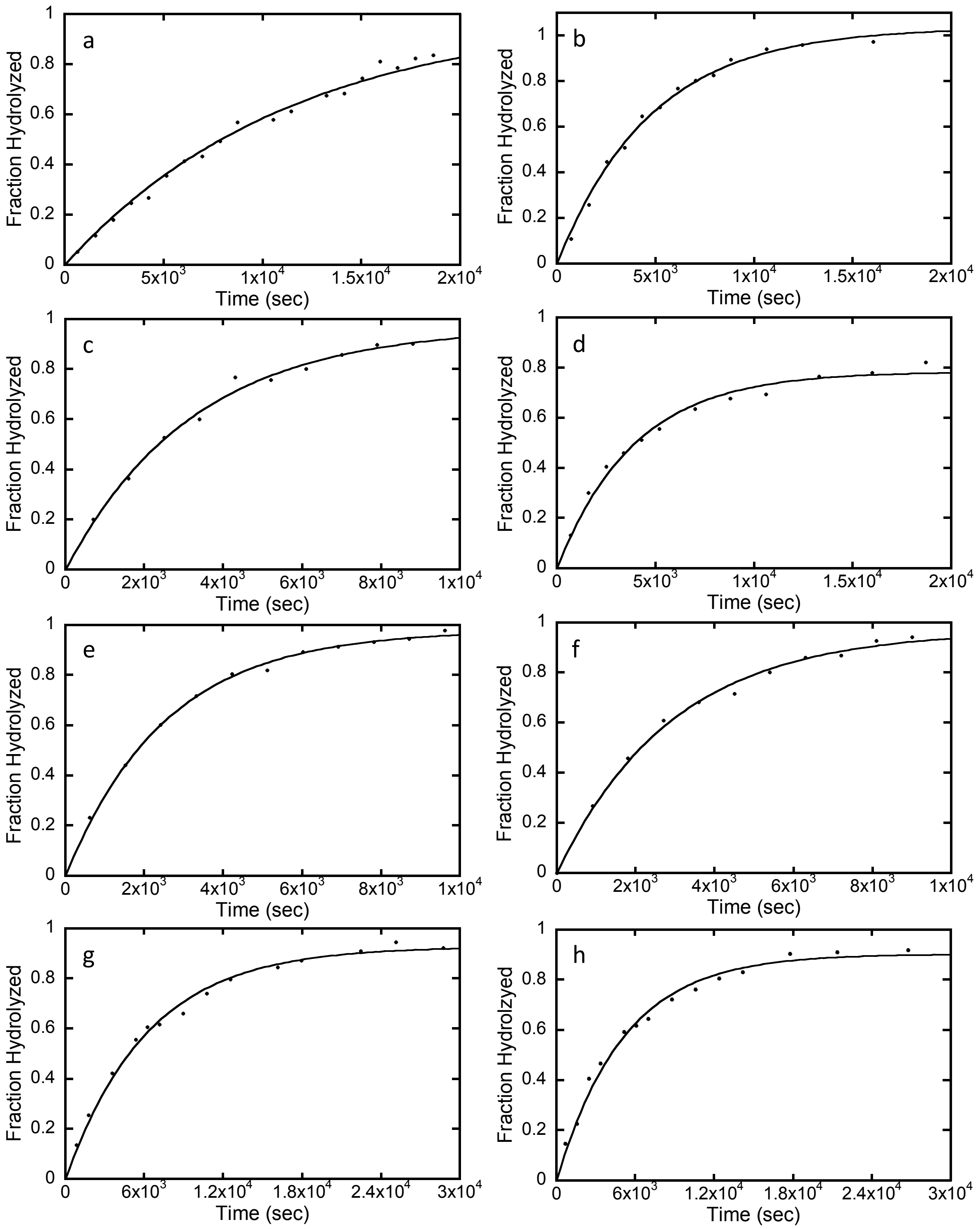

Figure S14: Time courses for the hydrolysis of compounds **3**-**10** by *Sy*-PhoK followed by ^{31}P NMR. Panel a shows hydrolysis of 2-chloroethyl phosphate (**3**). Panel b shows hydrolysis of 1,3-dichloroisopropyl phosphate (**4**). Panel c shows hydrolysis of 2-butoxyethyl phosphate (**5**). Panel d shows hydrolysis of butyl phosphate (**6**). Panel e shows hydrolysis of ethyl phosphate (**7**). Panel f shows hydrolysis of methyl phosphate. Panel g shows hydrolysis of glycerol phosphate (**9**, mixed isomers). Panel h shows hydrolysis of glucose-6-phsophate (**10**). All data are fit to equation 1 which yields the k_{cat}/K_m for the reaction.

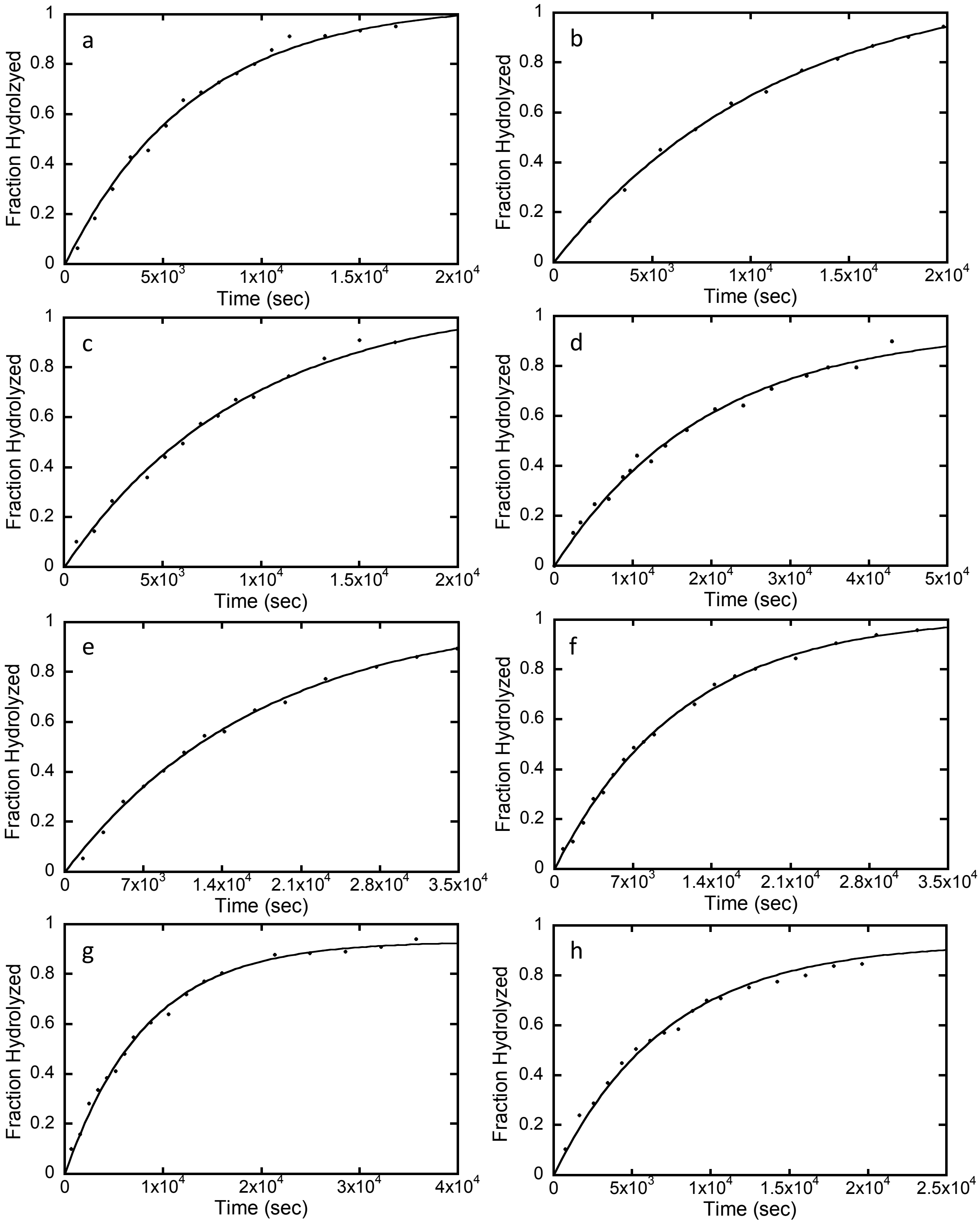

Figure S15: Time courses for the hydrolysis of compounds **3**-**10** by *Na*-PhoK followed by ^{31}P NMR. Panel a shows hydrolysis of 2-chloroethyl phosphate (**3**). Panel b shows hydrolysis of 1,3-dichloroisopropyl phosphate (**4**). Panel c shows hydrolysis of 2-butoxyethyl phosphate (**5**). Panel d shows hydrolysis of butyl phosphate (**6**). Panel e shows hydrolysis of ethyl phosphate (**7**). Panel f shows hydrolysis of methyl phosphate. Panel g shows hydrolysis of glycerol phosphate (**9**, mixed isomers). Panel h shows hydrolysis of glucose-6-phsophate (**10**). All data are fit to equation 1 which yields the k_{cat}/K_m for the reaction.

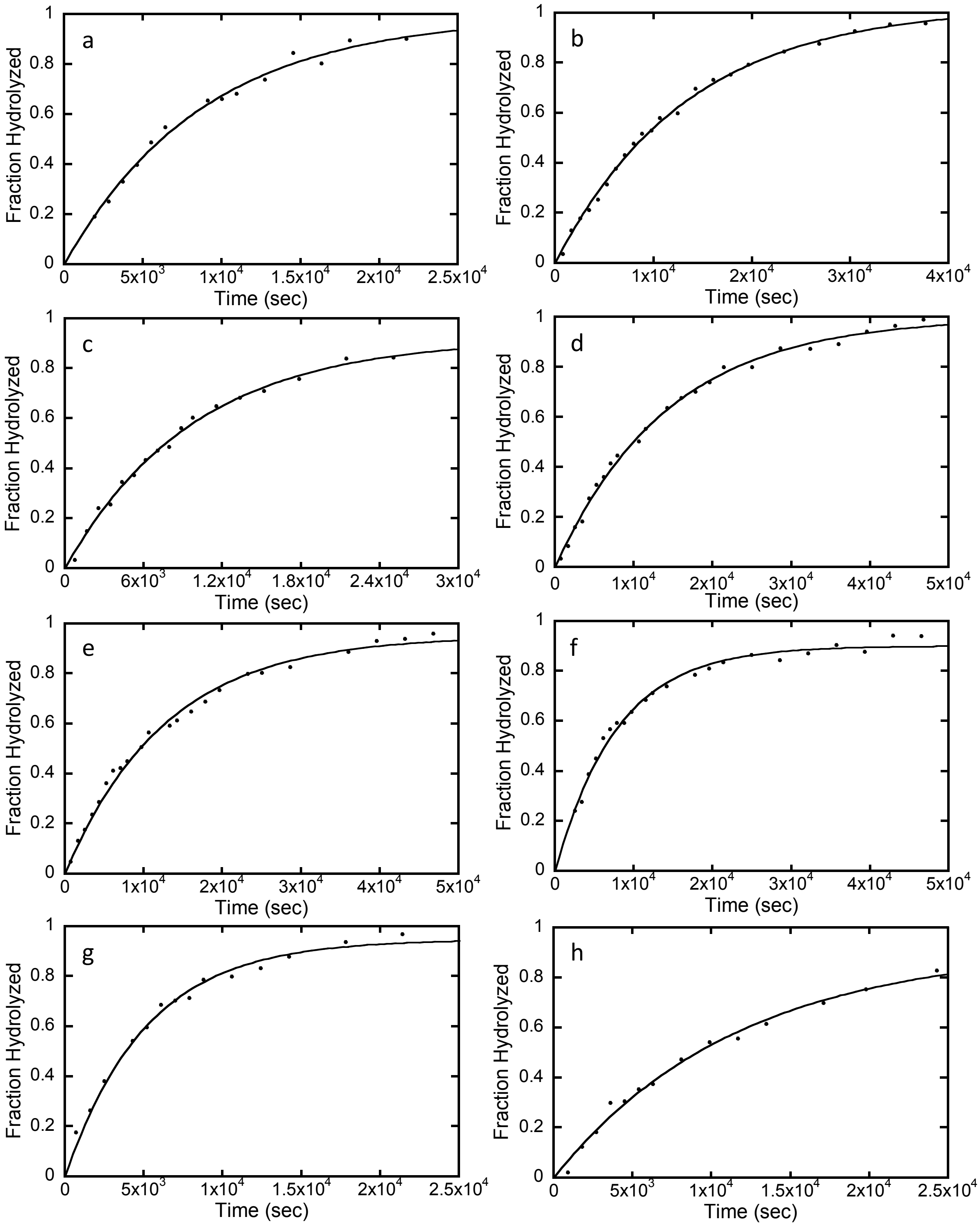

Figure S16: Time courses for the hydrolysis of compounds **3**-**10** by *No*-PhoK followed by ^{31}P NMR. Panel a shows hydrolysis of 2-chloroethyl phosphate (**3**). Panel b shows hydrolysis of 1,3-dichloroisopropyl phosphate (**4**). Panel c shows hydrolysis of 2-butoxyethyl phosphate (**5**). Panel d shows hydrolysis of butyl phosphate (**6**). Panel e shows hydrolysis of ethyl phosphate (**7**). Panel f shows hydrolysis of methyl phosphate. Panel g shows hydrolysis of glycerol phosphate (**9**, mixed isomers). Panel h shows hydrolysis of glucose-6-phsophate (**10**). All data are fit to equation 1 which yields the k_{cat}/K_m for the reaction.

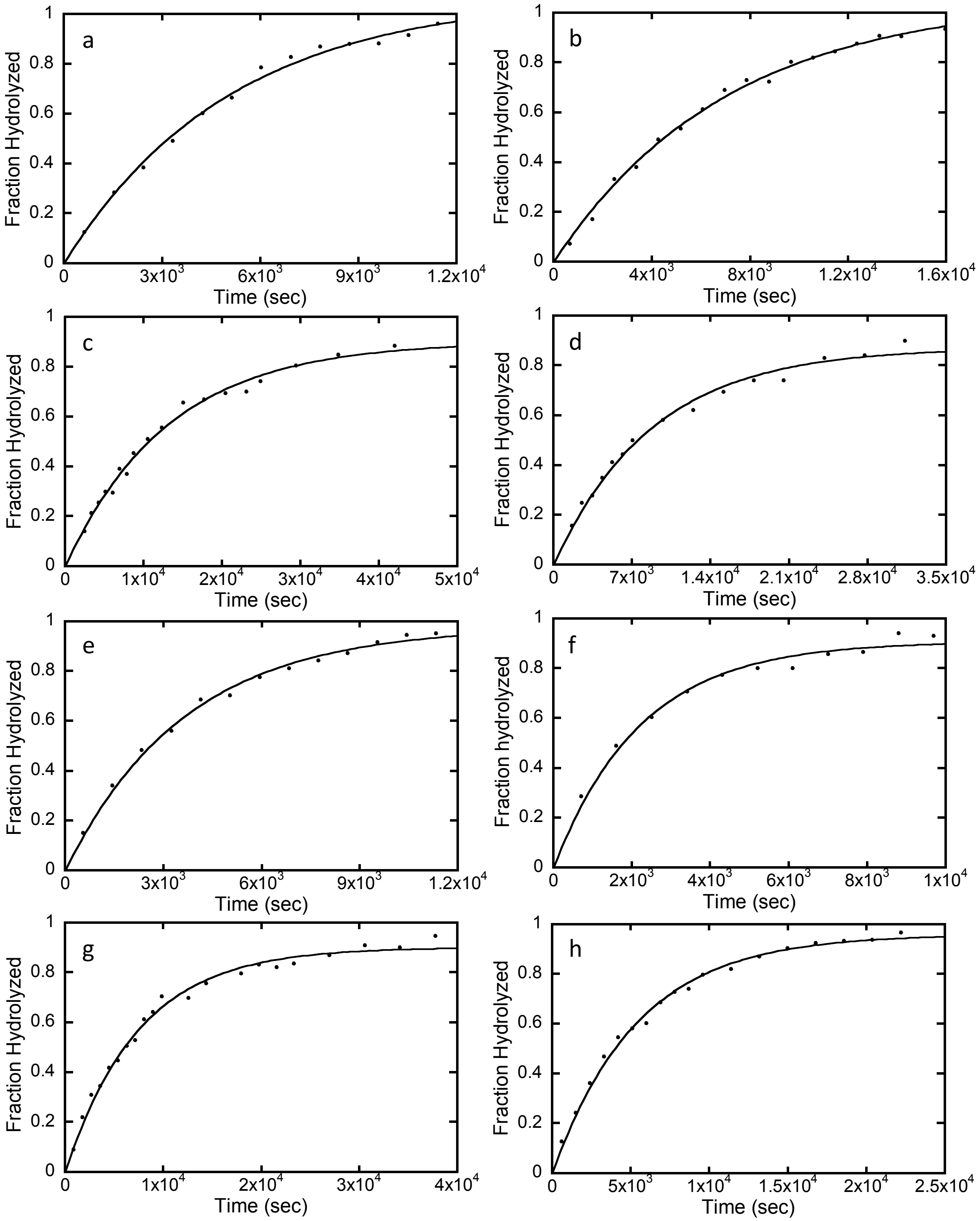

Figure S17: Time courses for the hydrolysis of compounds **3**-**10** by *Sm*-PhoK followed by ^{31}P NMR. Panel a shows hydrolysis of 2-chloroethyl phosphate (**3**). Panel b shows hydrolysis of 1,3-dichloroisopropyl phosphate (**4**). Panel c shows hydrolysis of 2-butoxyethyl phosphate (**5**). Panel d shows hydrolysis of butyl phosphate (**6**). Panel e shows hydrolysis of ethyl phosphate (**7**). Panel f shows hydrolysis of methyl phosphate. Panel g shows hydrolysis of glycerol phosphate (**9**, mixed isomers). Panel h shows hydrolysis of glucose-6-phsophate (**10**). All data are fit to equation 1 which yields the k_{cat}/K_m for the reaction.

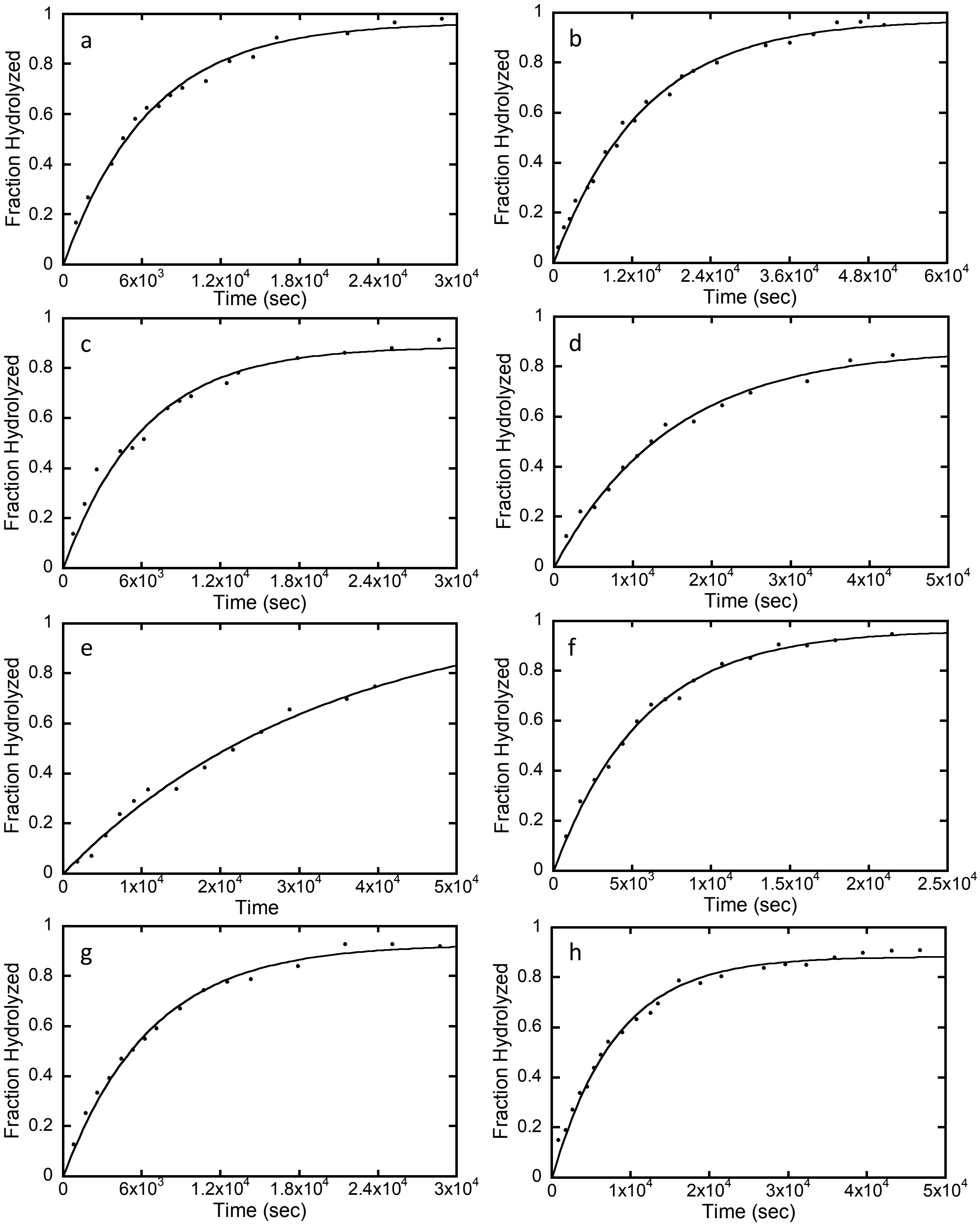

Figure S18: Time courses for the hydrolysis of compounds **3**-**10** by *Ng*-PhoK followed by ^{31}P NMR. Panel a shows hydrolysis of 2-chloroethyl phosphate (**3**). Panel b shows hydrolysis of 1,3-dichloroisopropyl phosphate (**4**). Panel c shows hydrolysis of 2-butoxyethyl phosphate (**5**). Panel d shows hydrolysis of butyl phosphate (**6**). Panel e shows hydrolysis of ethyl phosphate (**7**). Panel f shows hydrolysis of methyl phosphate. Panel g shows hydrolysis of glycerol phosphate (**9**, mixed isomers). Panel h shows hydrolysis of glucose-6-phsophate (**10**). All data are fit to equation 1 which yields the k_{cat}/K_m for the reaction.

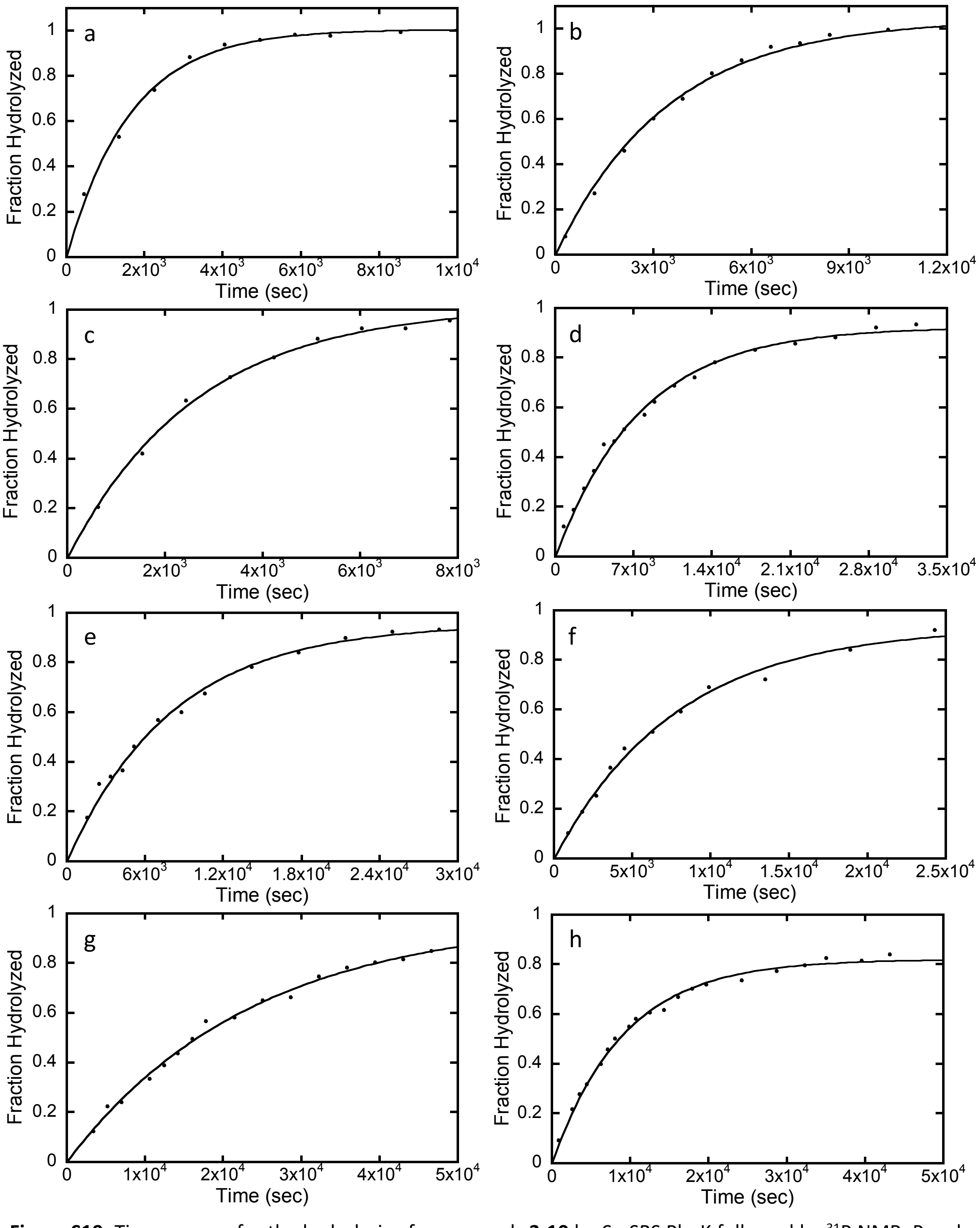

Figure S19: Time courses for the hydrolysis of compounds **3**-**10** by *SmSRS*-PhoK followed by ^{31}P NMR. Panel a shows hydrolysis of 2-chloroethyl phosphate (**3**). Panel b shows hydrolysis of 1,3-dichloroisopropyl phosphate (**4**). Panel c shows hydrolysis of 2-butoxyethyl phosphate (**5**). Panel d shows hydrolysis of butyl phosphate (**6**). Panel e shows hydrolysis of ethyl phosphate (**7**). Panel f shows hydrolysis of methyl phosphate. Panel g shows hydrolysis of glycerol phosphate (**9**, mixed isomers). Panel h shows hydrolysis of glucose-6-phsophate (**10**). All data are fit to equation 1 which yields the k_{cat}/K_m for the reaction.

Table S2. Kinetic constants and experimental conditions for homologs of *Sb*-PhoK with compounds **1** and **2**.

Enzyme	Substrate	k_{cat} (s^{-1})	K_m (μM)	k_{cat}/K_m ($M^{-1}s^{-1}$)	Enzyme Concentration	Max Substrate Concentration (mM)	Min Substrate Concentration (μM)
Sb-PhoK	4-nitrophenyl phosphate (**1**)	416 ± 3	69 ± 2	6.1 ± 0.2 x 10^6	0.165 nM	2.75	8.6
Sb-PhoK	phenyl phosphate (**2**)	460 ± 10	650 ± 30	7.1 ± 0.4 x 10^5	3.3 nM	2.32	7.25
*Sm*TDK1-PhoK	4-nitrophenyl phosphate (**1**)	460 ± 5	450 ± 10	1.02 ± 0.03 x 10^6	0.20 nM	2.75	8.6
*Sm*TDK1-PhoK	phenyl phosphate (**2**)	365 ± 7	600 ± 20	6.1 ± 0.3 x 10^5	2.0 nM	2	37.5
St-PhoK	4-nitrophenyl phosphate (**1**)	440 ± 5	78 ± 3	5.7 ± 0.2 x 10^6	0.13 Nm	2.75	8.6
St-PhoK	phenyl phosphate (**2**)	314 ± 6	81 ± 5	3.9 ± 0.2 x 10^6	1.8 nM	1.5	4.8
Sy-PhoK	4-nitrophenyl phosphate (**1**)	1230 ± 10	176 ± 4	7.0 ± 0.2 x 10^6	0.066 nM	2.75	12.9
Sy-PhoK	phenyl phosphate (**2**)	930 ± 30	670 ± 40	1.4 ± 0.1 x 10^6	0.63 nM	2.32	7.25
Na-PhoK	4-nitrophenyl phosphate (**1**)	303 ± 3	89 ± 3	3.4 ± 0.1 x 10^6	0.31 nM	2.75	8.6
Na-PhoK	phenyl phosphate (**2**)	336 ± 6	390 ± 20	8.7 ± 0.4 x 10^5	3.0 nM	2.32	7.25
No-PhoK	4-nitrophenyl phosphate (**1**)	621 ± 6	153 ± 5	4.1 ± 1.1 x 10^6	0.16 nM	2.75	8.6
No-PhoK	phenyl phosphate (**2**)	259 ± 6	300 ± 20	8.7 ± 0.6 x 10^5	3.2 nM	2.32	7.25
Sm-PhoK	4-nitrophenyl phosphate (**1**)	1370 ± 10	174 ± 4	7.9 ± 0.2 x 10^6	0.094 nM	2.75	12.9
Sm-PhoK	phenyl phosphate (**2**)	1100 ± 10	600 ± 20	1.84 ± 0.06 x 10^6	0.77 nM	2.32	7.25
Ng-PhoK	4-nitrophenyl phosphate (**1**)	297 ± 4	90 ± 4	3.3 ± 0.1 x 10^6	0.24 nM	2.75	8.6
Ng-PhoK	phenyl phosphate (**2**)	190 ± 3	250 ± 9	7.5 ± 0.3 x 10^5	2.3 nM	2.32	7.25
*Sm*SRS2-PhoK	4-nitrophenyl phosphate (**1**)	12.0 ± 0.1	88 ± 4	1.36 ± 0.07 x 10^5	11.3 nM	2.75	12.9
*Sm*SRS2-PhoK	phenyl phosphate (**2**)	16.6 ± 0.2	104 ± 5	1.59 ± 0.07 x 10^5	22.5 nM	2	6.25

Table S3. Experimental conditions and enzymatic efficiency of PhoK homologs from ^{31}P NMR kinetics experiments.

Enzyme	Substrate	Substrate concentration	Enzyme Concentration	k_{cat}/K_m ($M^{-1}s^{-1}$)
Sb-PhoK	2-chloroethyl phosphate (**3**)	2.5 mM	8.1 nM	$3.6 \pm 0.3 \times 10^4$
	1,3-dichloroisopropyl phosphate (**4**)	2.5 mM	4.1 nM	$1.65 \pm 0.09 \times 10^4$
	2-butoxyethyl phosphate (**5**)	2.5 mM	4.1 nM	$2.9 \pm 0.2 \times 10^4$
	butyl phosphate (**6**)	2.5 mM	8.1 nM	$1.33 \pm 0.08 \times 10^4$
	ethyl phosphate (**7**)	2.5 mM	4.05 nM	$2.7 \pm 0.2 \times 10^4$
	methyl phosphate (**8**)	1.25 mM	8.4 nM	$1.17 \pm 0.06 \times 10^4$
	glycerol phosphate (**9**)	2.5 mM	4.1 nM	$2.5 \pm 0.1 \times 10^4$
	glucose-6-phosphate (**10**)	2.5 mM	8.2 nM	$2.2 \pm 0.1 \times 10^4$
*Sm*TDK1-PhoK	2-chloroethyl phosphate (**3**)	2.5 mM	5.0 nM	$3.6 \pm 0.3 \times 10^4$
	1,3-dichloroisopropyl phosphate (**4**)	2.5 mM	2.5 nM	$5.4 \pm 0.3 \times 10^4$
	2-butoxyethyl phosphate (**5**)	2.5 mM	5.0 nM	$1.92 \pm 0.06 \times 10^4$
	butyl phosphate (**6**)	1.73 mM	20.0 nM	$4.2 \pm 0.2 \times 10^3$
	ethyl phosphate (**7**)	2.5 mM	10 nM	$1.08 \pm 0.04 \times 10^4$
	methyl phosphate (**8**)	2.5 mM	5.0 nM	$1.97 \pm 0.06 \times 10^4$
	glycerol phosphate (**9**)	2.5 mM	10 nM	$2.03 \pm 0.08 \times 10^4$
	glucose-6-phosphate (**10**)	2.5 mM	20.0 nM	$1.1 \pm 0.1 \times 10^4$
St-PhoK	2-chloroethyl phosphate (**3**)	2.5 mM	2.2 nM	$7.8 \pm 0.3 \times 10^4$
	1,3-dichloroisopropyl phosphate (**4**)	1.4 mM	3.3 nM	$2.34 \pm 0.09 \times 10^4$
	2-butoxyethyl phosphate (**5**)	2.5 mM	3.3 nM	$2.68 \pm 0.2 \times 10^4$
	butyl phosphate (**6**)	2.5 mM	6.5 nM	$1.21 \pm 0.04 \times 10^4$
	ethyl phosphate (**7**)	2.5 mM	6.5 nM	$1.72 \pm 0.04 \times 10^4$
	methyl phosphate (**8**)	2.5 mM	6.5 nM	$1.75 \pm 0.05 \times 10^4$
	glycerol phosphate (**9**)	2.5 mM	1.7 nM	$2.29 \pm 0.05 \times 10^4$
	glucose-6-phosphate (**10**)	2.5 mM	6.5 nM	$2.18 \pm 0.09 \times 10^4$
Sy-PhoK	2-chloroethyl phosphate (**3**)	2.5 mM	0.83 nM	$7.1 \pm 0.9 \times 10^4$
	1,3-dichloroisopropyl phosphate (**4**)	2.5 mM	2.1 nM	$1.00 \pm 0.05 \times 10^5$
	2-butoxyethyl phosphate (**5**)	2.5 mM	4.07 nM	$7.6 \pm 0.5 \times 10^4$
	butyl phosphate (**6**)	2.5 mM	8.3 nM	$3.1 \pm 0.2 \times 10^4$
	ethyl phosphate (**7**)	2.5 mM	8.3 nM	$4.8 \pm 0.1 \times 10^4$
	methyl phosphate (**8**)	2.5 mM	8.3 nM	$4.1 \pm 0.2 \times 10^4$
	glycerol phosphate (**9**)	2.5 mM	2.8 nM	$5.9 \pm 0.3 \times 10^4$
	glucose-6-phosphate (**10**)	2.5 mM	4.1 nM	$4.8 \pm 0.3 \times 10^4$
Na-PhoK	2-chloroethyl phosphate (**3**)	2.5 mM	3.87 nM	$4.1 \pm 0.2 \times 10^4$
	1,3-dichloroisopropyl phosphate (**4**)	1.0 mM	7.7 nM	$1.14 \pm 0.05 \times 10^4$
	2-butoxyethyl phosphate (**5**)	2.5 mM	3.87 nM	$3.2 \pm 0.1 \times 10^4$
	butyl phosphate (**6**)	2.5 mM	3.87 nM	$1.36 \pm 0.09 \times 10^4$
	ethyl phosphate (**7**)	2.5 mM	3.87 nM	$1.51 \pm 1.07 \times 10^4$
	methyl phosphate (**8**)	2.5 mM	7.7 nM	$1.14 \pm 0.03 \times 10^4$
	glycerol phosphate (**9**)	2.5 mM	6.4 nM	$1.91 \pm 0.06 \times 10^4$
	glucose-6-phosphate (**10**)	2.5 mM	7.7 nM	$1.81 \pm 0.09 \times 10^4$
No-PhoK	2-chloroethyl phosphate (**3**)	2.5 mM	1.84 nM	$6.2 \pm 0.5 \times 10^4$
	1,3-dichloroisopropyl phosphate (**4**)	2.5 mM	4 nM	$1.85 \pm 0.05 \times 10^4$
	2-butoxyethyl phosphate (**5**)	2.5 mM	1.84 nM	$5.6 \pm 0.3 \times 10^4$
	butyl phosphate (**6**)	2.5 mM	1.84 nM	$1.60 \pm 0.05 \times 10^4$
	ethyl phosphate (**7**)	2.5 mM	1.84 nM	$1.63 \pm 0.07 \times 10^4$
	methyl phosphate (**8**)	2.5 mM	4 nM	$1.59 \pm 0.05 \times 10^4$
	glycerol phosphate (**9**)	2.5 mM	8.0 nM	$2.4 \pm 0.1 \times 10^4$
	glucose-6-phosphate (**10**)	1.4 mM	8.0 nM	$1.07 \pm 0.09 \times 10^4$

Table S3 continued.

Enzyme	Substrate	Substrate concentration	Enzyme Concentration	k_{cat}/K_m ($M^{-1}s^{-1}$)
Sm-PhoK	2-chloroethyl phosphate (**3**)	2.5 mM	2.4 nM	$8.4 \pm 05 \times 10^4$
	1,3-dichloroisopropyl phosphate (**4**)	2.5 mM	3.12 nM	$4.5 \pm 0.2 \times 10^4$
	2-butoxyethyl phosphate (**5**)	2.5 mM	0.94 nM	$7.9 \pm 0.4 \times 10^4$
	butyl phosphate (**6**)	2.5 mM	4.7 nM	$2.42 \pm 0.1 \times 10^4$
	ethyl phosphate (**7**)	2.5 mM	9.4 nM	$2.9 \pm 0.1 \times 10^4$
	methyl phosphate (**8**)	2.5 mM	9.4 nM	$4.8 \pm 0.3 \times 10^4$
	glycerol phosphate (**9**)	2.5 mM	2.4 nM	$5.7 \pm 0.2 \times 10^4$
	glucose-6-phosphate (**10**)	2.5 mM	4.7 nM	$3.9 \pm 0.1 \times 10^4$
Ng-PhoK	2-chloroethyl phosphate (**3**)	2.5 mM	4.0 nM	$3.8 \pm 0.2 \times 10^4$
	1,3-dichloroisopropyl phosphate (**4**)	2.5 mM	2.0 nM	$3.7 \pm 0.1 \times 10^4$
	2-butoxyethyl phosphate (**5**)	2.5 mM	5.94 nM	$2.7 \pm 0.2 \times 10^4$
	butyl phosphate (**6**)	2.5 mM	5.94 nM	$1.16 \pm 0.07 \times 10^4$
	ethyl phosphate (**7**)	2.5 mM	5.94 nM	$5.4 \pm 0.9 \times 10^3$
	methyl phosphate (**8**)	2.5 mM	11.9 nM	$1.48 \pm 0.05 \times 10^4$
	glycerol phosphate (**9**)	2.5 mM	7.9 nM	$1.91 \pm 0.08 \times 10^4$
	glucose-6-phosphate (**10**)	2.5 mM	5.9 nM	$2.10 \pm 0.08 \times 10^4$
*Sm*SRS2-PhoK	2-chloroethyl phosphate (**3**)	2.5 mM	282 nM	$2.1 \pm 0.2 \times 10^3$
	1,3-dichloroisopropyl phosphate (**4**)	2.5 mM	282 nM	$1.00 \pm 0.09 \times 10^3$
	2-butoxyethyl phosphate (**5**)	2.5 mM	282 nM	$1.31 \pm 0.09 \times 10^3$
	butyl phosphate (**6**)	2.5 mM	282 nM	$4.4 \pm 0.2 \times 10^2$
	ethyl phosphate (**7**)	2.5 mM	282 nM	$4.4 \pm 0.2 \times 10^2$
	methyl phosphate (**8**)	2.5 mM	282 nM	$4.5 \pm 0.2 \times 10^2$
	glycerol phosphate (**9**)	2.5 mM	28.2 nM	$4.56 \pm 0.09 \times 10^3$
	glucose-6-phosphate (**10**)	2.5 mM	113 nM	$9.7 \pm 0.3 \times 10^2$

REFERENCES

[1] Balaban, R. S., and Ferretti, J. A. (1983) Rates of enzyme-catalyzed exchange determined by two-dimensional NMR: a study of glucose 6-phosphate anomerization and isomerization, *Proc Natl Acad Sci U S A 80*, 1241-1245.

[2] Bhinderwala, F., Evans, P., Jones, K., Laws, B. R., Smith, T. G., Morton, M., and Powers, R. (2020) Phosphorus NMR and Its Application to Metabolomics, *Anal Chem 92*, 9536-9545.
